# Systematic review of economic evaluations of varicella vaccination programmes

**DOI:** 10.1371/journal.pone.0282327

**Published:** 2023-03-27

**Authors:** Robert Anderson, Sungwook Kim, Nia Roberts, Stavros Petrou

**Affiliations:** 1 Centre for Health Service Economics and Organisation, Nuffield Department of Primary Care Health Sciences, University of Oxford, Oxford, United Kingdom; 2 Nuffield Department of Primary Care Health Sciences, University of Oxford, Oxford, United Kingdom; 3 Health Care Libraries, Bodleian Libraries, University of Oxford, Oxford, United Kingdom; Istituto Superiore di Sanità and St. Camillus International University of Health Sciences, ITALY

## Abstract

**Objectives:**

This study carried out a systematic literature review of economic evaluations of varicella vaccination programmes from the earliest publication to the present day, including programmes in the workplace and in special risk groups as well as universal childhood vaccination and catch up programmes.

**Methods:**

Articles published from 1985 until 2022 were sourced from PubMed/Medline, Embase, Web of Science, NHSEED and Econlit. Eligible economic evaluations, which included posters and conference abstracts, were identified by two reviewers who scrutinised each other’s selections at both title and abstract and full report stages. The studies are described in terms of their methodological characteristics. Their results are aggregated by type of vaccination programme and the nature of the economic outcome.

**Results:**

A total of 2575 articles were identified of which 79 qualified as economic evaluations. A total of 55 studies focused on universal childhood vaccination, 10 on the workplace and 14 on high risk groups. Twenty-seven studies reported estimates of incremental cost per quality-adjusted life year (QALY) gained, 16 reported benefit-cost ratios, 20 reported cost-effectiveness outcomes in terms of incremental cost per event or life saved and 16 reported cost-cost offset results. Most studies of universal childhood vaccination reported an increase in overall costs to health services, but often a reduction in cost from a societal perspective.

**Conclusions:**

The evidence surrounding the cost-effectiveness of varicella vaccination programmes remains sparse with contrasting conclusions in some areas. Future research should particularly aim to encompass the impact of universal childhood vaccination programmes on herpes zoster among adults.

## Introduction

Varicella-zoster is a highly infectious virus, which causes varicella (chickenpox), mainly as a mild self-limiting disease in young children, although it can lead to pneumonia and encephalitis in those with compromised immunity [1]. The virus remains in the body and can reactivate in the form of herpes zoster (HZ) (shingles) in later life. HZ is generally more serious and can lead to chronic neuropathic pain [2].

In England and Wales, the annual average number of cases of varicella was 670,866 in 1991–2000 of which 51% occurred in the 0–4 years age group and 15% in those aged 15 years and over. There were 275,268 general practitioner (GP) consultations, 2189 hospital admissions and 25 deaths (16% of which were in the under 15s). The corresponding figures for HZ were 244,818 cases, 2148 hospital admissions and 49 deaths, all but three in the over 65s [3]. In England and Wales, annual GP consultations for varicella fell by 22% in 1–3 year olds, and by 17% in infants, over the period 2004 to 2014 [4]. This fall may have reflected changes in accessibility and consulting behaviour. It does not necessarily indicate a reduction in disease; indeed, annual hospital admissions with varicella increased by 26% over a similar period (2004–16) [5].

An attenuated strain of varicella, the Japanese OKA-strain, is used in the production of varicella vaccines licensed in many countries worldwide. This vaccine was first licensed for high-risk children in several European countries in 1984, and its use was later extended to all children [6]. Several licensed formulations of live attenuated vaccines are currently available, as monovalent or combined with measles, mumps and rubella [7]. A single dose provides protection of 76%-85% in children [8]. Two doses provide 98% protection in children [9] and about 75% protection in adolescents and adults [10].

Thirty-six countries have a universal childhood vaccination programme [11]. They are widely spread geographically and among middle- and high-income countries. Many countries have no programme, including most countries in Northern and Eastern Europe. Concerns about a shift of varicella incidence to older age groups, where it results in more serious sequelae, and a reduction in exogenous boosting of protection against HZ, may have led some countries to decide against introducing a childhood vaccination programme. A review of the trends in countries with long standing programmes has shown substantial reductions in consultations and hospital admissions [12]. No definitive and consistent association has emerged between vaccination and an increase in HZ incidence in the elderly.

Economic evaluations of varicella vaccination have been published over recent years, mostly for routine childhood programmes. Systematic reviews of this literature have been published over the past two decades: Thiry et al (2003) included 17 studies [13], Rozenbaum et al (2008) 22 studies [14], Unim et al (2013) 15 studies [15], and Damm et al (2015) 38 studies in high income countries [16]. This systematic review aims to update the literature, to include not only childhood and catch up programmes but also vaccination of high risk groups and key staff, and to include all economic evaluations of varicella vaccination wherever and whenever published. Particular attention is paid to the usefulness of the outcome measures for policy making.

## Methods

Following PRISMA guidelines, we devised search terms which an expert research librarian used to conduct the search strategy (Appendix 1 in S1 File) [17]. We registered the systematic review with PROSPERO (number CRD42021249206) on 19 April 2021 [18].

The databases searched, on 24 April 2022, were: EconLit (1969-present), Embase (1974-present), MEDLINE (1946-present), NHS Economic Evaluation Database (inception to 31 March 2015), Science Citation Index, Social Science Citation Index and Conference Proceedings Citation Index (1900-present). Inclusion criteria were as follows: economic evaluation of varicella (chickenpox) vaccination programme regardless of type of economic evaluation (e.g. cost-effectiveness analysis, cost-utility analysis, cost-benefit analysis, cost-minimisation analysis or cost-consequences analysis) or vehicle (e.g. trial-based, decision model, etc). Conference abstracts were included as well as journal articles. Exclusion criteria encompassed studies that were: not an economic evaluation; not focused on varicella vaccination; review article not containing primary research evidence; or not published in the English language.

A two-tier screening process was implemented, whereby two health economists (RA and SWK) independently screened all titles and abstracts to identify relevant articles. Subsequently, eligible articles were fully screened, and data was extracted using bespoke data extraction forms (see Appendix 2 in S1 File). At any stage, disagreements between the reviewers were resolved by discussion, and if necessary, through consultation with the lead health economist (SP), to resolve uncertainties regarding study eligibility, or aspects of study design, conduct, analysis or reporting.

We categorised the data extracted as follows. Under strategy and methods, the subheadings were author, publication date, location, vaccination strategy (target population, dosage and comparator), methods (model type, whether herd immunity and HZ covered, time horizon, size and nature of the cohort), and characteristics of economic evaluation (outputs, perspective, discount rate). Under results, the sub-headings were delineated in terms of effects on cases, hospital admissions, mortality, costs and cost-effectiveness/utility/benefit with the results for the latter category further delineated by healthcare and/or societal perspective, and threshold analysis.

Cost changes were expressed in percentage terms. Otherwise monetary values were converted into 2021 £ sterling at purchasing power parity rates using the web-based Campbell and Cochrane Economics Methods Group (CCEMG) and the Evidence for Policy and Practice Information and Coordinating Centre (EPPI-Centre) cost converter [19].

The value of systematic literature reviews of economic evaluations is a subject of debate [20].

The studies identified here varied widely in matters such as the target groups, form of economic evaluation (cost-minimisation, cost-benefit, cost-effectiveness, cost-utility), the type of model applied (decision tree, Markov, dynamic transmission), the perspective taken (healthcare payer or society as a whole), the range of effects included (herd immunity, impact on herpes zoster, exogenous boosting), time horizon, discount rate, unit costs, vaccine price and other local inputs, and expression of economic outcomes. In view of these variations, we did not attempt to synthesise outputs by using meta-analysis. Nevertheless, we believe decision makers will find a narrative synthesis informative in identifying the range and quality of studies and in helping them to understand the structure of the resource allocation problem that they are addressing and the impact of the main parameters on the overall results [21, 22]. The Consensus on Health Economic Criteria (CHEC) [23] checklist was used to assess the methodological quality of the contributing studies and the Consolidated Health Economic Evaluation Reporting Standards (CHEERS) checklist [24] was used to assess their reporting quality.

The results are presented in terms of the key characteristics of the studies, followed by presentation by type of vaccination programme. For economic evaluations of universal childhood vaccination programmes, the results are further disaggregated by the nature of the economic outcome, e.g. incremental cost per quality-adjusted life year (QALY) gained, benefit-cost ratio, etc.

The project had the benefit of contributions from a Patient and Public Involvement team provided by the Patient and Public Involvement and Patient Experiences Programme at the RCN Research Institute, Warwick Medical School, University of Warwick.

## Results

**Fig 1:**
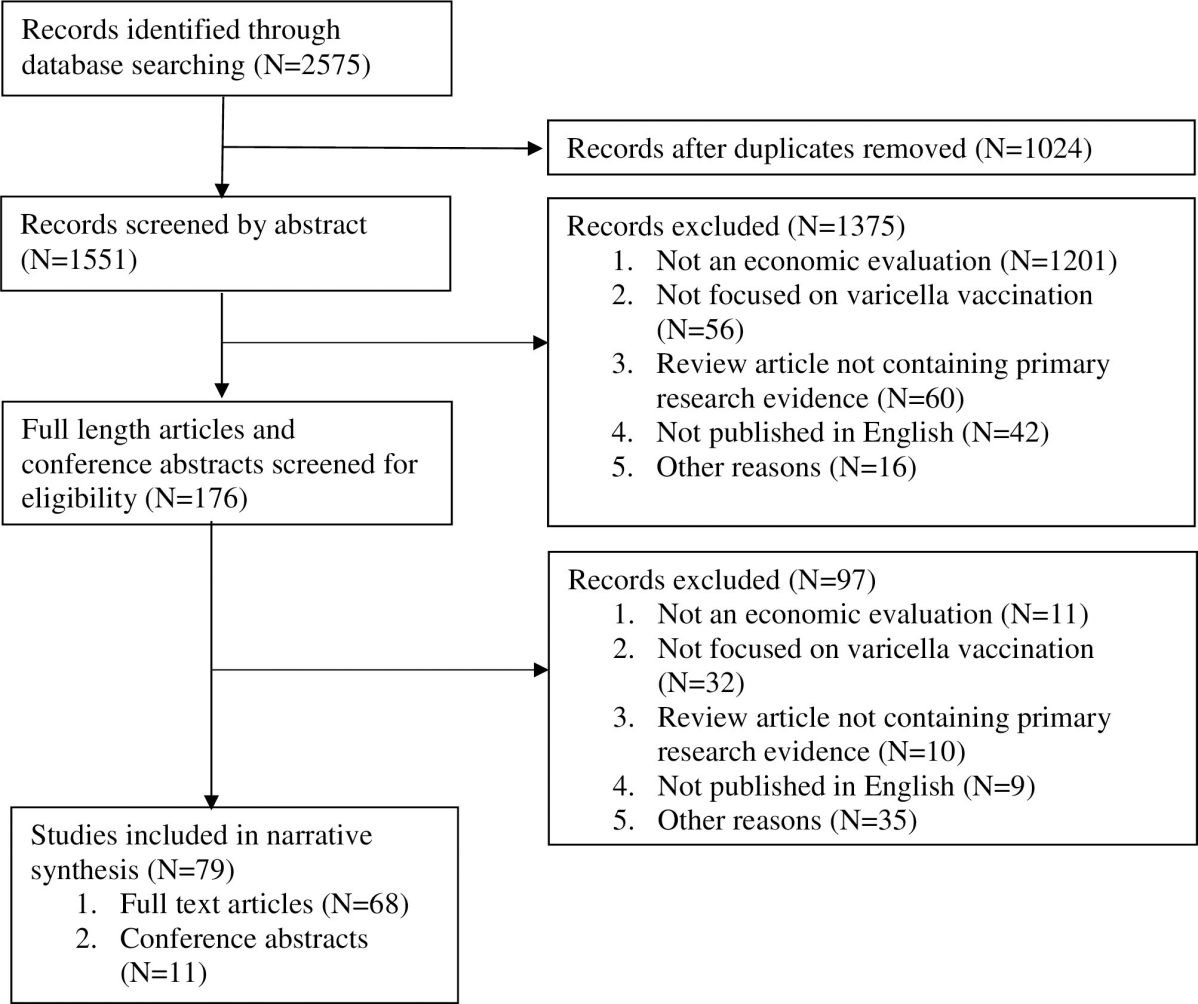
PRISMA flow diagram.

A PRISMA flow diagram summarising the results of the screening and assessment processes is presented in Fig 1. Of the 2575 records identified from the searches, 79 (68 full articles and 11 conference abstracts) survived the filtering process which removed duplicates, records which were not economic evaluations or not in the English language. The results are summarised in Table 1. Fuller details are set out in Appendix 3 of S1 File Table 1 (strategy and methods) and Table 2 (results) in S1 File.

**Table 1:** Summary of economic evaluations of varicella vaccination by setting and perspective. (Costs are presented in 2021 £ sterling unless otherwise stated).

	**Summary of economic evaluations of varicella vaccination in young children**			
			**Results**	
**Target population**	**Comparators**	**Settings**	**Healthcare perspective**	**Societal perspective**
Children at 12 months^77^	One dose vs no vaccination	Australia	£59/case averted^77^	n/a
Children aged 1 year and 4, 6 or 11^82^; at 1−2^91^	Two doses vs no vaccination^82,91^	Belgium	Effect of boosting on time to meet passmark^82^; BCR 0.67^91^	BCR 3.47^91^
Children at 12 months^102,103^	One dose vs some vaccination of vulnerable groups^102,103^	Brazil	£11,636/LYG^102,103^	£10,490/LYG^102,103^
Children at 13 months and 6 years^66^	Two doses vs no vaccination	Bulgaria	Dominates^66^	n/a
Children at 12 months^70,92^; at 12 months, 18 months and 4–6 years^55^	One dose^70,92^, two doses^55^ vs no vaccination	Canada	£39/case, £78,000/LYG (vaccination) £59/case, £39,000/LYG (catch up)^70^; BCR 0.61^92^; Cost/QALY £76,000^55^	Vaccination plus catch up dominates^70^; BCR 5.24^92^; Dominates^55^
Children 12 and 15 months^57^	One or two doses, universal or selected by serotesting	China	Cost/QALY £16,051 £31,968 £26,566 £38,555 depending on selection^57^	Satisfies GDP threshold^57^
Children eligible for other childhood vaccinations^65^; at 12–15 months^52^	One dose^65^, two doses^52^ vs no vaccination	Colombia	£2173/LYG^65^; £2200/LYG, £1100/DALY^52^	n/a
Children eligible for MMR^83,84^; at 12 months^99^; at 9 months to 6 years^101^	Dose not stated^83,84^; one dose ^99,101^ vs no vaccination	France	£14,575/QALY^83^; £3347/QALY^84^; 4% reduction in healthcare costs^99^; 16% reduction in healthcare costs^101^	Dominates^83,84^; 40% reduction in healthcare costs^101^
Children at 12 months^100^	One dose vs no vaccination	France/Germany	Reduction in healthcare costs: France 6.7%, Germany 51.1%^100^	Reduction in costs: France 59.6%, Germany 61.4%^100^
Children at 15 months^49,67^; those in current programme^50^; children at 12–18 months^94^	Two doses^49,67^, one dose^94^ vs no vaccination; current programme^50^	Germany	BCR 1.75^49^; 0.82 ^67^; 1.75^94^	BCR 4.12^49^; 4.6^67^; 4.12^94^; current programme is dominated by do nothing assuming exogenous boosting ^50^
Children at 12 and 15 months^58^	One or two doses vs no vaccination	Iran	n/a	£13,478/DALY (1 dose) £32,394/DALY (2 doses) cf 14,292/DALY GDP benchmark^58^
Susceptible children at 12 months old^72^	One dose vs no vaccination	Israel	BCR 1.63^72^	BCR 19.33^72^
Children at 15 months and 5–6 years^54^; infants^71^; children at 12–15 months and 5 to 6 years^81^; receiving MMR^85^; at 1–2 years^89^	Two doses^54,81,85^, one dose^71,89^ vs no vaccination	Italy	Dominates^54,81^; £14,866/QALY^85^; 16% reduction in healthcare costs^89^	38%^71^, 40%^89^ reduction in healthcare costs; Dominates^81^
Children at 12 months and 6 years^60^	One or two doses vs no vaccination	Mexico	Cost saving^60^	Cost saving^60^
Children at 12^68^ or 14 months and 4 years^87^	Two doses vs no vaccination^68,87^	Netherlands	£39,770/QALY^68^	£2787/QALY^68^; with boosting not cost-effective^87^
Children at 15 months^76^	Dose not reported vs current user pays situation	New Zealand	BCR 0.67^76^	BCR 2.79^76^
Children at 15 or 18 months and 7 or 11 years^62^	Two doses vs no vaccination	Norway	Dominates^62^	Dominates^62^
Children aged 12–18 months and 4 years^98^	One or two doses vs no vaccination	Peru	Cost saving^98^	n/a
Children at 12 or 15 months and 6 years^61^	Two doses vs no vaccination	Russia	£50,000/QALY^61^	Dominates^61^
Children at 12^80^, 15^93^ months, at 1 and 3 years^75^	Two doses vs no vaccination^75,80,93^	Spain	BCR 1.05^80^;0.54^93^	BCR 1.24^75^;2.67^80^;1.61^93^
Children at 12 and 18 months^88^	Two doses vs no vaccination	Sweden		Dominates^88^
Children at one and two years^90^, at 9–24 months^51^	Two doses vs routine vaccination of susceptible 11–15 year olds^90^, 10% coverage^51^	Switzerland	BCR 0.3^90^; £18,542 £18,204 £20,944/QALY^51^	BCR 1.29^90^; £15,219 £14,814 £17,472/QALY^51^
Healthy children at 15 months^95^; hypothetical birth cohort^97^	One dose^95^, dose not reported^97^ vs no vaccination	Taiwan	BCR 0.34^95^; 0.36^97^	BCR 2.06^95^; 1.44^97^
Children 12 months and 18 months or 6 years^53^	Two doses vs no vaccination	Turkey		Dominates^53^
Those eligible for childhood vaccinations ^56^, Children at 13 months and 3 years^59^, at 1 and 3 years^63^, at 12 months^64,86^	Two doses^56,59,63^, one dose^64^, not reported^86^ vs no vaccination	UK	41% of simulations met 20,000/QALY^56^; £5665/QALY^59^; £7267/QALY^63^; dominated^64^; £1674 £1033 £3187/QALY^86^	Dominates^59^
Children eligible for current programme^69^, at 15 months^73^, under 6 years^74^, at 12–15 months^78,79^, at 15 months^96^	Impact vs no impact of vaccination on asthma^69^; One dose^73,74,78^, two doses^79^_,_ dose not reprted^96^ vs no vaccination	US	177% increase incosts^73^; BCR 0.4^74^,0.3^89^; all childhood vaccinations together BCR 5.3^78^,3.0^79^	Net cost savings not sensitive to impact on asthma^69^;95% reduction in costs^73^;BCR 5.4^74^,6.9^96^; all childhood vaccinations together 16.5^78^,10.1^79^
	**Summary of economic evaluations of varicella vaccination in the workplace**			
			**Results**	
**Target population**	**Comparators**	**Settings**	**Employer perspective**	**Societal perspective**
Recruits to armed forces^42^	Two doses vs no vaccination^42^	India	£3404/case prevented £341/man day saved^42^	£5744/QALY^42^
Nurses and doctors under 45 tested for susceptibility^44^	Two doses vs no vaccination^44^	Israel	Incremental cost per case averted (with full testing for susceptibility) £22.8, (with partial testing) £198.4, vaccinate all £9837^44^	n/a
Susceptible army recruits^40^	One dose vs no vaccination^40^	Singapore	12% cost saving^40^	n/a
Hospital staff tested for suceptibiliy^46^; Paediatric staff^48^	Two doses vs no vaccination^46,48^	UK	Incremental cost per case averted (with full testing for susceptibility) £0.0, (with partial testing) £12-£18, vaccinate all £67^46^; 35% reduction in cost^48^	n/a
Officer cadets on entry^39^; army recruits^41^; all new hospital employees^43^; all hospital employees, those caring for patients, those in high risk situations^45^; healthcare workers^47^. Tested for susceptibility^39,41,43,45^	Two doses vs no vaccination^39,41,43,45,47^	US	72% reduction in cost^39^; incremental cost per case prevented by different testing regimes £900 £400 £2300^41^; cost per case prevented £40,300 £65,200 £75,300 with different testing regimes, vaccinate all £341,000^43^;with testing £16,000 vaccinate all £41,000^45^; reduction in cost per employee with testing £24 vaccinate all £65^47^	n/a
	**Summary of economic evaluation of varicella vaccination in special vulnerable groups, eg, immigrants, patients undergoing procedures**			
			**Results**	
**Target population**	**Comparators**	**Settings**	**Healthcare perspective**	**Societal perspective**
Paediatric transplant patients^29^; newly arrived adult immigrants and refugees^35^	One dose vs no vaccination^29,35^	Canada	Cost savings per child £3167^29^	Cost savings per child £3508^29^; Selective vaccination and none both dominated, ICER of vaccinate all vs serotesting £8220/QALY^35^
Susceptible adults 15−45^27^	Two doses vs no vaccination^27^	France	Cost per case avoided £43,964 (with history taking) £489 (with serotest)^27^	Cost per case avoided £43,384 (with history taking) negative (with serotest)^27^
Paediatric patients exposed to varicella virus and not indicated for VZIG prophylaxis^31^	One dose vs no vaccination^31^	Hong Kong	Dominates^31^	n/a
Susceptible 11 yearolds^26^; susceptible children adopted from abroad 1–18 years old^32^	One dose^26^, two doses^32^ vs no vaccination	Italy	BCR 0.20 (vaccinate all) 0.54 0.42 0.68 (test for susceptibility first)^26^; Cost per case prevented £21,391 (with serotesting) £25,452 (vaccinate all)^32^	BCR 0.78 (vaccinate all) 0.54 0.42 0.68 (test for susceptibility and vaccinate susceptibles) 2.17 1.16 2.17^26^
All newly arriving asylum seekers age 15−39^33^	Vaccination of all susceptible in response to outbreak. Two doses vs usual response^33^	Switzerland	Cost per person 167% higher^33^	n/a
Primigravidae born in UK or Bangladesh^37^	Two doses vs no vaccination	UK	Verbal screening followed by serotesting may be cost-saving to the NHS for both UK- and Bangladesh-born primigravidae^37^	n/a
Susceptible schoolchildren 6–12 and adolescents 13−17^25^; 20 year olds and older^28^; susceptible children eligible for kidney transplant^30^; recently arrived refugees 1 to 20 years of age^34^; women 15–49 postpartum^36^; women postpartum^38^	One dose vs no vaccination^25^; two doses vs no vaccination^28^; Pre-treatment vaccination two doses vs usual care^30^; One dose under 13, two doses 13 and over vs no vaccination^34^; two doses vs no vaccination^36^; Dosage not reported vs no vaccination^38^	US	Cost per case prevented £150 (children) £289-£562 (adolescents)^25^; ICER £7200-£17m/QALY dependent on age and testing regime^28^; Cost per case—reduction of 88%^30^; Cost per case prevented £1272-£3566 depending on testing regime^36^; Screening and vaccination option dominates^38^	Cost per case prevented savings (children) £105-£388 (adolescents)^25^; ICER dominates-£17m/QALY dependent on age and testing regime^28^; Cost minimising to vaccinate children 1–4 without testing, to test first other ages^34^; Cost per case prevented dominates or £1528 depending on testing^36^

BCR–Benefit:cost ratio

ICER–Incremental cost effectiveness ratio

LYG–Life-year gained

MMR–Measles, mumps and rubella vaccine

QALY–Quality-adjusted life-year

n/a–not applicable

### Characteristics of studies

Eighteen high income countries dominated the settings (Australia, Belgium (2), Canada (5), France (6), Germany (5), Hong Kong, Israel (2), Italy (7), Netherlands (2), Norway, New Zealand, Singapore, Spain (3), Sweden, Switzerland (3), Taiwan (2), UK (8), US (17)), with only twelve of the 79 studies from ten middle income countries (Brazil (2), Bulgaria, China, Colombia (2), India, Iran, Mexico Peru, Russia, Turkey). Studies have been published at a steady rate since 1995: before 1990 (1), 1990–1994 (3), 1995–1999 (11), 2000–2004 (17), 2005–2009 (14), 2010–2014 (10), 2015–2019 (12), 2020 (3), 2021 (5), 2022 (3). The majority of studies, 55, focused on universal childhood vaccination at about 12–15 months (and up to 6 years), some adding a catch up. Other target groups in which universalist policies were studied were susceptible school age children [25], susceptible 11 year olds [26], and susceptible adults 15–45 [27, 28].

There were studies in high risk or otherwise special groups: those eligible for organ transplants–children [29] and adults [30]; paediatric patients [31]; recent immigrants, refugees or children adopted from abroad [32–35]; susceptible adults [28] and children aged six or over [25]; adolescents [26], other adults [27]; and women pre- or post-partum [36–38]. Ten studies focused on the workplace, four on military personnel [39–42] and six on healthcare staff [43–48].

The comparator was usually no vaccination, although in three studies it was the vaccination programme in force [49–51]. Where there was more than one option, some studies undertook incremental analysis in such a way that each option was compared with the one immediately below it in order of cost, after identifying and eliminating dominated options. Among such studies which reported incremental cost per QALY estimates, there were five full examples [35, 52–55] and three partial [28, 56, 57]. Six others with more than one option presented enough information to follow this procedure but did not do so [51, 58–62].

Policies targeting susceptibles may elect to identify them or simply vaccinate without testing. There are two tests, history-taking and serological testing and various combinations. A common option was to take a history, then serotest those with a negative history of vaccination or infection and vaccinate those testing susceptible. Some studies analysed several options. Many of these did not present their results in the form of a cost-effectiveness frontier. Figueira et al (2003) presented a formula for the prevalence of antibody above which it is more cost-effective to test for antibody than to vaccinate without testing [34]. Of studies with more than two options, three performed appropriate incremental analysis [25, 28, 41], five did not [26, 36, 43, 46, 47], and in three it was not clear [31, 37, 63].

Results were reported from two broad perspectives, the healthcare payer or society as a whole. In practice, the societal perspective involved adding an estimate of the value of production loss entailed by carers taking time off work to look after a sick child. However, since 2003 some studies have considered the patient’s perspective measured by QALY loss suffered by a sick child [64]. Studies in the workplace and those targeted at special risk groups took the payer’s or employer’s perspective only, except for one which reported QALYs gained by vaccinees [42]. Most of the others reported both perspectives. Of the studies of universal childhood vaccination, 13 reported from the payer’s perspective only, 8 from the societal perspective only and 32 from both.

Two basic types of model were used in analysis of varicella vaccination: static and dynamic. Static models usually took the form of decision analysis. They could not take account of herd immunity, an increase in the average age of infection (when it is more serious), or HZ, mediated by external boosting. Age structured transmission dynamic models do take account of these effects. The latter were confined to childhood vaccination programmes. However, 21 studies of childhood vaccination programmes used a static model [52, 54, 57, 58, 60, 61, 65–79]. No study targeting any other group used a dynamic model.

Common forms of economic evaluation are cost minimisation, cost-effectiveness, cost-utility and cost-benefit analysis [22]. In the studies reviewed here, a report of benefit-cost ratio (BCR) was common where the ratio was expressed as the change in hospital and other economic costs divided by the costs of vaccination. Many studies reported more than one type of economic metric. Twenty-seven studies reported estimates of incremental cost per QALY gained, all but three of which evaluated childhood vaccinations [31, 42, 80].

Among studies reporting a BCR, the most common pattern was a BCR below one from the payer’s perspective but greater than one from the societal perspective

### Results by type of vaccination programme by type of outcome reported

#### Universal childhood vaccination

The majority of studies, 53, focused on universal childhood vaccination at about 12–15 months for the first dose, up to 6 years for the second if any. Thirty-four used a dynamic model, 21 of which reported incremental cost per QALY values [27, 28, 50, 51, 53–56, 59, 62–64, 68, 81–88]. These studies tended to be the most recently published, with an average publication date of 2015.

To address current concerns in childhood vaccination, a range of issues were covered, singly or in various combinations: the effect of taking account of HZ [47, 50, 53, 54, 56, 59, 63, 81–83, 87, 88], the effect of boosting [50, 59, 81], age at second dose [53, 55], confining a universal programme to susceptible adolescents [64], or post exposure prophylaxis [31]. Some of the studies measuring QALYs focused on simpler issues: a standard programme without herd immunity or HZ [68], with a package including catchup [63], with measles, mumps and rubella (MMR) vaccine [84, 85] or a simple two dose childhood programme [81].

#### Universal childhood vaccination: Studies reporting incremental cost per QALY.

Estimates of incremental cost per QALY gained varied widely, over a range of factors, and cannot always be summarised as a single value. Some studies reported raw values in terms of incremental cost per QALY gained [53, 58, 59, 63, 64, 68, 83–86]. Others, having compared incremental cost per QALY results with a local threshold of acceptable cost per QALY, reported whether the threshold had been satisfied or not [51, 53, 56, 58, 59, 61, 64, 68, 87]. Yet others reported whether there was a dominant option in the sense of having a higher level of QALYs and lower cost than a comparator [16, 53, 54, 57, 59, 62, 65, 81, 83, 84, 88]. There were examples of disparate findings emerging from studies which apparently have much in common [59, 64].

A key issue in the more recent studies was the inclusion of effects of childhood vaccination on incidence of HZ in the elderly. Brisson et al (2003) reported that not vaccinating dominates universal childhood vaccination from the healthcare and societal perspectives, though a programme in 11 year old susceptibles would be marginal in relation to local thresholds [64]. Akpo et al (2020), on the other hand, reported an incremental cost per QALY gained of £5665 from the healthcare perspective, which would fully satisfy local thresholds, and dominate from a societal perspective [59]. In most of the other studies claiming to take account of it, the effect of HZ did not invariably compromise the outcome of childhood vaccination. We review these studies in publication order starting with the most recent. Heininger et al (2021), Pawaskar et al (2021) and Wolff (2021) reported that taking account of the effects of herpes zoster would not compromise the cost-effectiveness of varicella vaccination [51, 62, 88], whereas Rafftery et al (2021) reported that, on certain assumptions, it might [55]. Azzari et al (2020) reported that universal childhood vaccination dominates [81]. Wolfson et al (2019) reported that a two dose programme would meet a local cost-effectiveness threshold [53]. Melegaro et al (2018) reported that a programme of HZ vaccination rescued the childhood varicella programme, the joint cost per QALY of both programmes being £12,000 from a healthcare perspective, probably satisfying local cost-effectiveness thresholds [54]. Van Lier et al (2015), however, agreed with Brisson et al [64]: with immune boosting, vaccination at 95% coverage was not cost-effective (threshold €20k per QALY) from a societal perspective [87]. In the study by Holl et al (2015), childhood vaccination achieved a modest incremental cost per QALY estimate of £7267 from a healthcare perspective [63]. In a conference abstract, Damm et al (2015) was pessimistic: discontinuation of an existing universal childhood programme would lead, irrespective of additional HZ vaccination, to both cost-savings and QALY gains when considering exogenous boosting [50]. Bilcke et al (2013) showed that results depended on assumptions about exogenous boosting and other assumptions in a complicated pattern [82]. Van Hoek et al (2012) studied a programme of joint HZ and childhood varicella vaccination and found that 50% of simulations met the local threshold [56]. Sauboin et al (2012) reported a cost-effectiveness estimate of £14,866 per QALY gained from a healthcare perspective [85]. Littlewood et al (2008) reported a similar cost-effectiveness estimate of £14,575 per QALY gained [83]. Gialoretti et al (2005) and Coudeville et al (2004) merely reported the reduction in varicella costs [71, 89].

Five other studies reported results in terms of incremental cost per QALY gained [52, 58, 68, 84, 86]. Two studied the choice between one and two doses, one with both options against a no vaccination comparator reported £13,478 for one dose, £32,394 for two [58]; the other reported an incremental cost per disability-adjusted life year (DALY) of £1100 for one dose and £15,300 for two doses against a one dose comparator [52]. One study explored different analytical approaches, but the outcomes were consistently in the range of £1674-£3147 per additional QALY [86]. An outcome on a similar scale was reported in a comparison of quadrivalent MMR plus varicella vaccine (MMRV) with MMR vaccine: £3347 and £3248 with a catch up [84]. A high incremental cost of £39,770 per QALY from the healthcare perspective was reported in another study, though the societal counterpart was much lower - £2787 [68].

#### Universal childhood vaccination: Studies reporting benefit-cost ratios

Fifteen studies reported BCRs [49, 67, 72, 74–76, 80, 90–97], of which only four reported positive ratios from the healthcare perspective [49, 67, 72, 94]. Three of these were set in Germany where the health insurer provides an allowance to parents off work to look after a sick child (Kinderkrankengeld), which is included in the healthcare perspective [49, 67, 94]. In other studies, the value of time off work was included in the societal perspective. In the fourth study, a vaccine price substantially below the average for the studies reviewed accounts for its exception to the rule [72]. Finally, BCRs from the societal perspective were universally positive.

These fifteen studies have an average publication date of 2002. In all but two, the intervention in the infant option was a single dose vaccine [90, 91]. They mostly considered three options; no vaccination, infant vaccination, and a catch up in 2–11 year olds or a routine programme in susceptible adolescents. The adjuncts to the childhood programme were usually evaluated bundled in with it. A study in Switzerland took the programme in force, vaccinating susceptible adolescents, as the comparator, and considered substituting or adding a childhood programme [90]. These options recorded BCRs of 0.3 and 0.27, respectively, from a healthcare perspective. In other studies, the usual pattern was that the option with the adjunct to the childhood programme had a lower BCR, whether the BCR was below or above one [49, 67, 80]. In one study, a free standing programme in susceptible adolescents slightly outperformed a childhood programme either by itself or with a catch up (0.73 vs 0.61 and 0.6, respectively) [92]. The single option studies consistently reported BCRs below one from the healthcare perspective (0.54 [93], 0.34 [95], 0.9 [74], 0.3 [96], 0.67 [76], 0.36 [97]) apart from those set in Germany where Kranklengeld makes all the difference [49, 67, 94], and the one set in Israel with a very low vaccine price [72].

#### Universal childhood vaccination: Studies reporting in terms of cost saving or cost per event prevented

Studies reporting cost savings are equivalent to a benefit-cost ratio, provided that the cost of vaccination is included. There were eight such studies [69, 71, 73, 89, 98–101]. However, it is uncertain whether vaccination costs were included in these studies. Four studies reported incremental cost per life year gained or incremental cost per death prevented, but not incremental cost per QALY gained or a benefit-cost ratio [65, 70, 77, 102, 103]. Since death, though not unknown, is uncommon in varicella, we would expect these costs to be high. However, three of the studies reported fairly modest estimates of incremental cost per life year gained– £2173 [65], £28,000 [70], and £11,636 [103]. The incremental cost per death prevented was estimated at £9.6 million in the study that reported it [77].

### Universal childhood vaccination: Catch ups

Sixteen studies of childhood programmes also evaluated a catch up in children from the age of two up to adolescence. All were set in high income countries [25, 49, 63, 64, 67, 70, 71, 77, 80, 82, 84, 89, 91, 92, 99, 100]. Nine assumed tests to identify susceptibles [25, 49, 64, 67, 70, 71, 77, 91, 92]. The results reflected the heterogeneity of outcome reporting seen in the evaluations of the universal childhood programmes. Most of the catchups performed unfavourably, but there is no hard and fast rule. There were reports of low incremental cost per case prevented (£59) [70], incremental cost per QALY gained (£3284) [84] and a benefit-cost ratio exceeding one [49].

### Special high risk groups

Seven studies focused on identifying susceptible adolescents or adults for vaccination where a universal childhood programme was not in force [25–28, 36–38]. These studies did not take account of herd immunity or any impact on herpes zoster, effects likely to be negligible from these interventions. The studies differed in terms of outcome reported. The BCR of vaccinating 11 year olds was below one from a healthcare perspective [26]. The incremental cost per QALY gained from vaccinating susceptible 20–29 years olds was £7200 (£167,000 in adults 30 and over) [28]. The incremental cost per case prevented was estimated at £150 in children and £289-£562 in adults [25], and £389 in 15 year olds [38]. The incremental cost per case prevented was estimated at £1272 for the mother and £472 for the baby when postpartum women were studied [36].

Immigrants and refugees generally have greater susceptibility than the host population and should benefit from vaccination, especially if closely confined. Four studies have focused on these groups [32–35]. Two were modelling studies, one a before and after study, and another a clinical examination. In three, the decision question was presumptive vaccination versus testing first. One provided a formula for determining the choice [34]. Another showed an incremental cost of £394 per case prevented with testing [32]. A third reported an incremental cost per QALY gained of £10,816 for presumptive vaccination against testing and vaccinating susceptibles [35]. A study of outbreak control in housing facilities for asylum seekers comparing vaccination following an outbreak with the usual response reported that vaccination is more costly but more effective, but no composite cost-effectiveness outcome was offered [33].

Vaccination is an option in children eligible for organ transplants in whom post-transplant infection would entail costly intervention. Two studies showed that it would, on balance, save hospital costs [29, 30]. Post exposure vaccination of paediatric patients contra-indicated for varicella zoster immune globulin (VZIG) also led to lower costs and also averted QALY losses [31].

### Working environments

There were a few economic evaluations of vaccination programmes in the workplace, notably for health care workers–to avoid costly furloughing in response to an outbreak; and military recruits–to avoid disruption to training schedules, or military personnel–to avoid absence of a team member. These studies often evaluated policies of testing for susceptibility and confining vaccination to susceptibles. They had a short time horizon; in the case of healthcare staff, this was restricted to the average length of service at a particular site. A before-and-after study was sometimes used. The perspective was the employer and the focus was on the net cost. The perspective of the vaccinee was considered in only one study [42].

In the US, testing and vaccinating army personnel resulted in an incremental cost per case prevented of £400 [41]. In Singapore, there was a cost saving of £1.58 per vaccine, including cases prevented and service days gained [40]. A programme in US Air Force cadets resulted in an incremental saving of £59 per person screened [39]. A programme in recruits to the Indian armed forces was deemed cost-effective on the strength of an incremental cost per QALY of £5744.

Because of close contact with infected patients, healthcare staff are at increased risk of infection. Six studies evaluated staff vaccination programmes, mostly testing then vaccinating susceptibles. All but one were modelling studies. Values of incremental cost per case prevented were £40,300 (new recruits) [43], £22,800 (doctors and nurses under 45) [44], or £16,000 (all employees) [45]. Two studies reported other outcomes–an incremental saving of £721 per incident (of exposure to infection) prevented [46], or an incremental saving of £24 per person vaccinated with a policy of vaccinating without testing [47]. A cross-section audit of vaccination across 22 hospitals led the authors to conclude that “it is likely that vaccination represents a cost-effective intervention” but they did not quantify their cost-effectiveness estimates [48].

### Patient and public involvement

Our PPI team adopted the view that complications of chickenpox had not received due weight by the contributing economic evaluations. It was noted that none of the contributing studies had had the benefit of PPI.

## Discussion

### Summary

This systematic review of economic evaluations of varicella vaccination programmes identified 79 studies covering a range of target groups and jurisdictions. Our review adopts a narrative format as heterogeneity precluded meta-analysis of economic outcomes. Fifty-five studies focused on universal infant programmes. The natural course of a varicella epidemic is complicated by herd effects and possible effects on HZ. This requires dynamic modelling, something that distinguishes recent studies from earlier ones, and in effect supersedes earlier methods. A key issue in the studies was perspective, healthcare only or societal including valuation of carers’ lost production. Studies did not include population preferences for patient outcomes until measures of their QALY gain became available in 2003.

The studies targeting special groups or workplaces–adults, primigravidae, new immigrants from low income countries, patients eligible for organ transplants, healthcare workers, military personnel–required, and received, less complicated modelling than childhood programmes as the effects on herd immunity and HZ are negligible in these groups. In all of these groups, a proportion may not be susceptible to infection and will not benefit from vaccination because they have had chickenpox or been vaccinated before. Whether to test for susceptibility, if so by what modalities, and confining vaccination to susceptibles is an issue in assessing the cost of intervention in these decision contexts.

### Discussion of results

Some general results have emerged from our systematic review. At current vaccine prices, a routine childhood vaccination programme would not save health service resources, but would recoup overall economic costs if the value of forgone production from carers’ time off work were included. Estimates of incremental cost per QALY gained were sometimes favourable in relation to local cost-effectiveness thresholds but sometimes not, depending on local inputs and handling of the effects on HZ. Adjuncts to a one dose routine childhood programme, e.g. two doses, catch up programme, etc., generally showed less favourable cost-effectiveness results. The current key issue is the impact on HZ, which has not been resolved. Testing for capacity to benefit is generally worthwhile.

Some measures of value for money are more informative than others. A positive BCR from the healthcare perspective establishes good value for money on the proviso that all the omitted factors would enhance it. The key omitted favourable factor in many studies is the gain in production from reduced carer time off work. However, BCRs typically do not include any allowance for the healthcare costs of HZ. A negative BCR from the healthcare perspective is not decisive against intervention. Few healthcare interventions spare healthcare resources on balance, and should be justified or otherwise by their overall impact in incremental cost-effectiveness terms. Estimates of incremental cost per event prevented is a partial measure as it requires an external measure of the value of preventing the clinical event of interest. The standard approach now is to assess estimates of incremental cost per QALY gained against a local cost-effectiveness threshold. Estimates of costs and QALYs should include herd effects and any effects on HZ. Whether or not to include effects related to carers remains an unsettled question [104].

### Comparison with other reviews

The two earliest reviews included studies up to 2007 [13, 14]. A more recent study covered universal vaccination during 1999–2008 [15]. Another focused on modelling studies, set in high income countries, which have considered the link between universal varicella vaccination and HZ [16]. As well as bringing coverage up to date (36 studies since 2008), the present review is more comprehensive with no restrictions to date or location of publication, target group, modelling method, or reporting metrics. A recent study appraised specific modelling challenges in six studies—so we have not covered this particular task [105].

### What this study adds

This systematic review describes all the economic evaluations of varicella vaccination programmes that have been carried out to date and collects them in a single source. Unusually for such reviews of economic evaluations, it also includes conference abstracts and posters. This proved fortunate as many of the most recent modelling studies, clearly using the most advanced methods, were reported in this format. The review assesses the strengths and weaknesses of the different forms of economic evaluation used in different contexts and highlights the importance of perspective in the usually binary decision of whether to adopt a programme or not. It also shows the importance of correct deployment of the ICER when assessing the acceptability of options such as adjuncts to a basic programme. In these ways, the review serves as a resource for researchers and decision makers.

### Strengths

In view of the international variation in the adoption of childhood vaccination policies and pressures on current non-adopters, this systematic review is timely in taking stock of all the available evidence and in assessing the variability in the findings and its sources. We undertook systematic comprehensive searches on the relevant databases from their inception, screened the studies, extracted the key data on a consistent basis and presented the results tables using a common and readable format.

### Limitations

First and foremost, in view of the heterogeneity of contributing studies, it was not possible to carry out a meta-analysis. Secondly, the text above highlights selected findings, inevitably involving some judgment by the authors, whereas many of the studies examine several options. However, fuller results are presented in Table 2 in S1 File. Thirdly, the results have been taken at face value with no attempt to probe their validity, for example, by replication where enough data was available. The one exception is the BCR findings, most of which proved to be based on the correct definition, but in two cases with enough data, the BCR could not be replicated [93, 97]. Fourthly, because of our inclusive selection criteria, many of the studies examined have been assessed in other systematic reviews. Nevertheless, we may have missed what may be important studies in languages other than English and have not tried to locate relevant grey literature.

### Implications for research and policy

The issue of the cost-effectiveness of universal childhood vaccination when HZ is taken into account is unresolved. It is unlikely to be resolved until the routine programmes have been in force for long enough to establish their effects on HZ.

As an interim step towards reconciling the various studies, it would be informative to develop a model that could subsume each of the others as a special case and use it to partition the differences in outcome between studies among factors such as inputs, assumptions, model parameters and model structure. This overarching approach could highlight areas for further scrutiny.

Other things being equal, the benefit of a universal childhood varicella vaccination programme depends on the burden of the disease, including the number of cases and the cost of treating these cases. The cost depends on the on the number of children and does not vary with the number of cases. Accordingly, in countries with as yet no programme it is worth maintaining surveillance of consultations and hospital admissions for any change which would alter the cost-effectiveness of a programme. Moreover, decision-makers in these countries could take stock of the trends in the burden of HZ in countries with long standing childhood varicella programmes for evidence as to the effects on HZ.

## Conclusions

This paper presents a systematic review of economic evaluations of varicella vaccination programmes. These studies have increased in scope and sophistication over time, so that the more recent studies are the more reliable. A key issue now appears to be the impact of varicella vaccination programmes on herpes zoster. This issue is likely to be illuminated by monitoring trends in countries with long-standing childhood vaccination programmes.

## Supporting information

S1 File Search strategy in Medline, Embase, Web of Science and other databases; data extraction form headings; analysis of contributing studies. (DOCX)

## Abbreviations

BCR: Benefit-cost ratio
CEA: Cost-effectiveness analysis
CUA: Cost-utility analysis
DALY: Disability-adjusted life-year
GDP: Gross domestic product
ICER: Incremental cost-effectiveness ratio
HZ: Herpes zoster (shingles)
MMR: Measles, mumps and rubella vaccine
MMRV: Varicella vaccine with MMR
NHS: United Kingdom National Health Service
PPI: Patient and Public Involvement
QALY: Quality-adjusted life-year
WHO: World Health Organisation
