# Supplementary material for: Systematic review of economic evaluations of varicella vaccination programmes
Source: PLoS One. 2023 Mar 27;18(3):e0282327. doi: 10.1371/journal.pone.0282327 (PMC10042376; doi:10.1371/journal.pone.0282327)
Supplement: S1 File — (DOCX) [file pone.0282327.s001.docx]

**Supplementary Material**

**S1 Appendix 1: Search strategy in Medline, Embase, Web of Science and other databases**

Medline:

|  | Medline (Ovid MEDLINE® Epub Ahead of Print, In-Process & Other Non-Indexed Citations, Ovid MEDLINE® Daily and Ovid MEDLINE®) 1946 to April 2022 |  |
| --- | --- | --- |
| 1 | Chickenpox Vaccine/ | 2198 |
| 2 | Chickenpox/ | 7741 |
| 3 | (varicella* or chicken pox or chickenpox).ti,ab,kw. | 17349 |
| 4 | 2 or 3 | 18419 |
| 5 | exp Immunization Programs/ | 15537 |
| 6 | exp immunization/ or exp vaccination/ | 196639 |
| 7 | (vaccin* or immuni?ation*).ti,ab,kw. | 425094 |
| 8 | 5 or 6 or 7 | 493180 |
| 9 | 4 and 8 | 5421 |
| 10 | 1 or 9 | 5669 |
| 11 | Economics/ | 27450 |
| 12 | exp "costs and cost analysis"/ | 257520 |
| 13 | Economics, Dental/ | 1920 |
| 14 | exp economics, hospital/ | 25559 |
| 15 | Economics, Medical/ | 9196 |
| 16 | Economics, Nursing/ | 4013 |
| 17 | Economics, Pharmaceutical/ | 3063 |
| 18 | (economic$ or cost or costs or costly or costing or price or prices or pricing or pharmacoeconomic$).ti,ab. | 938586 |
| 19 | (expenditure$ not energy).ti,ab. | 34024 |
| 20 | value for money.ti,ab. | 1955 |
| 21 | budget$.ti,ab. | 32964 |
| 22 | 11 or 12 or 13 or 14 or 15 or 16 or 17 or 18 or 19 or 20 or 21 | 1099344 |
| 23 | ((energy or oxygen) adj cost).ti,ab. | 4514 |
| 24 | (metabolic adj cost).ti,ab. | 1599 |
| 25 | ((energy or oxygen) adj expenditure).ti,ab. | 27520 |
| 26 | 23 or 24 or 25 | 32600 |
| 27 | 22 not 26 | 1091832 |
| 28 | 10 and 27 | 572 |
| 29 | exp animals/ not humans/ | 4999748 |
| 30 | 28 not 29 | 569 |

Embase:

|  | Embase 1974 to April 2022 |  |
| --- | --- | --- |
| 1 | chickenpox vaccine/ | 5137 |
| 2 | chickenpox/ | 12197 |
| 3 | (varicella* or chicken pox or chickenpox).ti,ab,kw. | 21632 |
| 4 | 2 or 3 | 25287 |
| 5 | exp *immunization/ | 128082 |
| 6 | (vaccin* or immuni?ation* or immuni?e?).ti,ab,kw. | 510319 |
| 7 | 5 or 6 | 524997 |
| 8 | 4 and 7 | 7970 |
| 9 | 1 or 8 | 9645 |
| 10 | Health Economics/ | 34207 |
| 11 | exp Economic Evaluation/ | 332699 |
| 12 | exp Health Care Cost/ | 317423 |
| 13 | pharmacoeconomics/ | 8809 |
| 14 | (economic$ or cost or costs or costly or costing or price or prices or pricing or pharmacoeconomic$).ti,ab. | 1215151 |
| 15 | (expenditure$ not energy).ti,ab. | 46032 |
| 16 | value for money.ti,ab. | 2685 |
| 17 | budget$.ti,ab. | 43340 |
| 18 | 10 or 11 or 12 or 13 or 14 or 15 or 16 or 17 | 1494959 |
| 19 | ((energy or oxygen) adj cost).ti,ab. | 4765 |
| 20 | (metabolic adj cost).ti,ab. | 1714 |
| 21 | ((energy or oxygen) adj expenditure).ti,ab. | 34815 |
| 22 | 19 or 20 or 21 | 40128 |
| 23 | 18 not 22 | 1486872 |
| 24 | 9 and 23 | 1264 |
| 25 | (exp animals/ or nonhuman/) not human/ | 6939985 |
| 26 | 24 not 25 | 1247 |

Web of Science Core Collection:

| # 3   \|  \| \| --- \| | [688](http://ezproxy-prd.bodleian.ox.ac.uk:2249/summary.do;jsessionid=12A2441AE7ADC0C255EA75EC60CAC1F8?product=WOS&doc=1&qid=4&SID=E1Ti3tHkUC2rkrM5PCG&search_mode=CombineSearches&update_back2search_link_param=yes) | #2 AND #1 |
| --- | --- | --- | --- |
| # 2 | [3,088,778](http://ezproxy-prd.bodleian.ox.ac.uk:2249/summary.do;jsessionid=12A2441AE7ADC0C255EA75EC60CAC1F8?product=WOS&doc=1&qid=3&SID=E1Ti3tHkUC2rkrM5PCG&search_mode=GeneralSearch&update_back2search_link_param=yes) | **TOPIC:** (economic* or cost or costs or costly or costing or price or prices or pricing or pharmacoeconomic* OR "value for money" OR budget*)  *NOT*  **TOPIC:**  ("energy cost*" OR "oxygen cost*" OR "metabolic cost*" OR "energy expenditure" OR "oxygen expenditure") |
| \| # 1 \| \| --- \| | [5,486](http://ezproxy-prd.bodleian.ox.ac.uk:2249/summary.do;jsessionid=12A2441AE7ADC0C255EA75EC60CAC1F8?product=WOS&doc=1&qid=1&SID=E1Ti3tHkUC2rkrM5PCG&search_mode=GeneralSearch&update_back2search_link_param=yes) | **TOPIC:**  ("chicken pox" OR chickenpox OR varicella*)  *AND*  **TOPIC:**  (vaccin* OR immunisation* OR immunization* OR immunise* OR immunize*) |

EconLit:

| noft("chicken pox" OR chickenpox OR varicella*) AND noft(vaccin* OR immunisation* OR immunization* OR immunise* OR immunize*) | 15 |
| --- | --- |

NHSEED:

| ((chicken pox OR chickenpox OR varicella*) AND (vaccin* OR immunisation* OR immunization* OR immunise* OR immunize*)) and ((Economic evaluation:ZDT and Bibliographic:ZPS) OR (Economic evaluation:ZDT and Abstract:ZPS)) | 56 |
| --- | --- |

**S1** **Appendix 2: Data extraction form headings**

Publication details

Sources of funding

Author affiliation

Competing interests

Publication type

Setting and location

Patient characteristics

Type of intervention

Tests

Comparator intervention(s)

Cohort

Eligibility criteria

Study perspective

Type of economic evaluation

Analytical approach

Model used (if applicable)

Time frame of the analysis (time horizon)

Discount rate(s) for costs and outcomes

Currency, price date and conversion factors

Type and category of costs

Data source(s) for resource use

Methods for identifying resource use

Assumptions surrounding the measurement of resources

Costs results

List effects of intervention covered

Vaccine Coverage

Data source(s) for effects

Methods of measurement of effects

Methods of valuation of effects

Report health effects

Cost-effectiveness/cost-benefit outcome(s)

Parental work days lost per case, value of work day lost

Analyses to address uncertainty, heterogeneity and distributional effects

Results of analyses that address uncertainty, heterogeneity and distributional effects

Authors’ conclusions

Quality score based on CHEERS and CHEC checklists

Report and describe any patient and public involvement (PPI)

Comments of person extracting the data

**S1 Appendix 3: Analysis of contributing studies**

**S1 Appendix 3: Table 1 Contributing studies: strategy and methods: location, target population, doses: model type and coverage: methods of economic evaluation: outputs and perspective etc**

|  | | Vaccination Strategy | | Methods: Model type and coverage | | | | | Methods: Economic evaluation | | |
| --- | --- | --- | --- | --- | --- | --- | --- | --- | --- | --- | --- |
| Author,  date | Location | Target  Population | Dosage;  comparator | Model type | Herd immun-ity | Herpes Zoster | Time horizon | Cohort,  size, nature | Outputs | Perspective | Discount rate pa |
| Akpo 2020^59^ | UK | Children 13 months and 3 years 4 months. | 2doses  1^st^ V or MMRV; 2nd MMRV  No vaccination  MMRV-varicella vaccination along with measles, mumps, rubella vaccination | Dynamic, adapted from a published model | yes | yes | Up to 100 years | Hypothetical; successive birth cohorts | Cost/QALY  ICER | Healthcare and societal | 3.5%  costs and benefits |
| Azzari  2020^81^ | Italy | Children 12-15 months and 5 to 6 years old. | 2 doses  No vaccination | Dynamic transmission model | yes | yes | 50 years | Single; hypothetical  birth cohort | Cost/QALY | Healthcare and societal | 3% costs and benefits |
| Banz  2003^49^ | Germany | 15 month old healthy children;  susceptible adolescents (11–12 years of age) | 2 doses  1: 15 month olds  2: 11-23 year olds  No vaccination | Age-structured, dynamic decision-analytical  model: EVITA. | yes | no | Up to 30 years | Single; hypothetical  birth cohort | Benefit:cost ratio;  ICER in terms of cost per event prevented, | Healthcare and societal | 5% costs  life years gained not discounted |
| Banz  2009^90^ | Switzerland | Children 1-2; susceptible adolescents 11-15 | 2 doses  Children 1-2  Routine vaccination of susceptible adolescents 11-15 | Age-structured, dynamic decision-analytical  model: EVITA. | yes | no | Up to 50 years | Single; hypothetical  birth cohort | Benefit-cost ratio; ICER in terms of cost per life year gained | Healthcare and societal | 5% costs  life years gained not discounted |
| Baracco 2015^43^ | United States;  Hospital | All new employees or those who test susceptible by clinical screening H or serology S; or S given H-. | 2 doses  No Vaccination | Decision tree | no | no | 8.9 years  (average duration of employment) | Single;  Hypothetical cohort of new employees | ICER: cost per case prevented | Healthcare (Employer) | None |
| Beutels 1996^67^ | Germany | 15 month old healthy children; susceptible 12 year olds | 2 doses  (booster after 10 years)  No Vaccination | Markov model | no | no | 70 years | Single; hypothetical  birth cohort | Benefit:cost ratio;  ICER:  cost per event prevented. | Payer and societal | 5% costs and benefits |
| Bilcke 2013^82^ | Belgium | Children aged 1 year and 4, 6 or 11  Adults 50 or 60 (HZ) | 1 dose at 1 year;  1 dose at 4,6, or 11.  No Vaccination | An existing age-structured transmission-dynamic model | yes | yes | Up to 100 years | Series of birth cohorts; hypothetical | Cost utility: incremental cost per QALY | Payer and losses to patients and carers in QALYs. | Costs: 3%  QALYs: 1.5% |
| Blas 2017^98^ | Peru | Children aged 12-18 months and 4 years old | 1 or 2 doses  1 dose  A: 12m B :18m  2 dose  C: A+18m  D: A+4y  No Vaccination | Dynamic transmission model | Not reported | Not reported | Implicitly at least 10 years | Not reported | Cost-cost | Payer | Not reported |
| Boccalini 2020^32^ | Italy | Susceptible children adopted from abroad 1-18 years old | 2 doses for those with negative serology;  2 without testing;  2 doses for those with negative or uncertain history  No vaccination | Decision tree | none | none | Implicitly up to age 18 | Series of birth cohorts; hypothetical | Cost effectiveness | Payer | None |
| Bonanni 2008^91^ | Belgium | Children 1-2  Adolescents 13 years old | 2 doses  1. children 1-2  2. adolescents 13  3. 1 plus susceptible adolescents  No vaccination | Age-structured, dynamic decision-analytical  model: EVITA | yes | none | 30 years | Single: birth cohort; hypothetical | Benefit:cost ratio | Healthcare and societal | 3% costs  0% health effects |
| Brisson 2002^92^ | Canada | Children at 1,5, 11 and 12 years old | 1 dose  1. children 1  2. 1 & 5-11 catch-up  3. preteen 12  No vaccination | Realistic age-structured deterministic  models (RAS) | yes | yes | 30 years | Single: birth cohort; hypothetical | Benefit:cost ratio: cost effectiveness ratio: cost per life year gained | Healthcare and Societal (includes loss of leisure time as well as time off work) | 3% costs and outcomes |
| Brisson Edmunds 2003^64^ | UK | Children 1-11 | 1 dose  1. children 1  2. 1&catch-up 2-11 in first year  3. adolescent: 11 year old susceptibles  No vaccination | A realistic age structured (RAS) transmission  dynamic model. | yes | yes | 80 years | Single: birth cohort; hypothetical | Cost per QALY.  Net cost to payer | Healthcare (payer) and societal | 3% costs and outcomes |
| Burnham 1998^39^ | United States Air Force Academy | Officer cadets on entry | 2 doses  V, SV, HV, HSV  V – vaccinate  S – serology  H – health history  No vaccination | Prediction of outcome in each option followed by implementation of SV then before and after study. | no | no | 1 year | Annual intake at USAF Academy  1400 | Cost-cost | Employer | Not applicable: short term study. |
| Chacon-Cruz 2022^60^ | Mexico | Children 1 and 6 | A: one dose at one year  B: two doses at 1 and 6  C: one dose at 1, one dose MMRV at 6  D: two doses MMRV at 1 and 6  No vaccination | Simple model factoring burden by  vaccine efficacy  times coverage | no | no | 20 years | Hypothetical; 20 successive birth cohorts | Incremental cost per case averted; incremental cost per life year gained | Healthcare and societal | 3% costs and benefits |
| Chodick 2005^44^ | Israel  Hospitals | Nurses and doctors under 45 | 2 doses  HSV, SV, V  No vaccination  HSV- sequence: H – take history, S – then serotest +ves,  then V - vaccinate seropsitives | Markov model | no | no | 20 years | Hypothetical cohort representing healthcare staff in Israel | Cost per case avoided. | Employer | 3% applied to cases |
| Chui 2014^31^ | Hong Kong | Paediatric patients exposed to varicella virus and not indicated for VZIG prophylaxis. | 1 dose  No vaccination | Decision tree | no | no | Very short | Hypothetical cohort: per average patient. | Cost and QALY separately before and after | Healthcare and patient | None.  Short study. |
| Coudeville 1999^101^ | France | Children 9 months to 6 years | 1 dose  No vaccination | Nonlinear, age-structured, deterministic model based on a set of partial differential equations. | no | no | 30 years | Population divided into 75 cohorts of identical size at birth; hypothetical | Cost-benefit analysis | Direct healthcare costs.  Indirect effects on work absence (not evaluated in money terms.) | 5% costs only |
| Coudeville 2003^99^ | France | Children aged 12 months; children age 6; children age 2-11. | 1 dose  Children age 12 months.  Catch up: six year programme for 6 year olds; first year vaccination of 2-11 year olds.  No vaccination | Epidemiological model. | no | no | 50 years | Not reported | Change in medical and social security costs | Public sector | Not reported |
| Coudeville 2004^89^ | Italy | Children 1-11 | 1 dose  1. Children 1-2  2. 1& catch up for 6 year olds  3. 1&children 2-11 in first year of programme  No vaccination | Nonlinear, age-structured, deterministic model based on a set of partial differential equations. | yes | no | 50 years | Population divided into 100 cohorts of identical size at birth; hypothetical | Change in costs | Health service and societal | 3% costs |
| Coudeville 2005^100^ | France and Germany | Children 1-11 | 1 dose  1. Children 1-2  2. 1&children 2-11 in first year of programme;  No vaccination | Nonlinear, age-structured, deterministic model based on a set of partial differential equations. | yes | no | 50 years | Population divided into 100 cohorts of identical size at birth; hypothetical | Change in costs  Cost effectiveness  (cost per life year) | Health service and societal | 3% costs and outcomes |
| Damm 2015^50^ | Germany | Those currently eligible for varicella vaccination | 2 doses  Discontinuation of current programme. Current programme | Age-structured dynamic model of varicella transmission and vaccination. | Not reported | yes | 100 years | Not specified | Change in costs and QALYs | Societal | 3% costs and outcomes |
| De Boer 2013^68^ | Netherlands | Children 14 months and 4 years old | 2 doses  No vaccination | Static cohort model | no | no | 30 years | Birth cohort of 180,000 | Cost-utility  Cost per QALY | Health payer and societal | none |
| De la Hoz 2011^65^ | Colombia | Children eligible for other childhood vaccinations | 1 dose  with possible ten year boosters  No vaccination | Decision analysis model | no | no | 30 years | Birth cohort | Cost effectiveness  Cost per life year gained | Healthcare payer | 3% costs and outcomes |
| De Valliere 2011^33^ | Vaud, Switzerland | All newly arriving asylum seekers age 15-39. | 2 doses  Vaccination of all susceptible in response to outbreak;  Usual response | Before and after study | no | no | Before: 8 months; after:15 months | 858 before; 966 after | Change in costs  Change in number of events | Payer | n/a |
| Diez Domingo 1999^93^ | Spain | Children 15 months old | 1 dose  No vaccination | Markov decision tree | no | no | 20 years | Single cohort; hypothetical | Cost-benefit analysis | Healthcare and societal | 5% costs  (only costs considered) |
| Dimitrova 2018^66^ | Bulgaria | Children 13 months and 6 years old | 1 or 2 doses  No vaccination | Dynamic transmission  model | no | no | 100 years | Not reported  Probably birth cohort | Cost effectiveness | Payer | Not reported |
| Ditkowski 2016^69^ | United States | Those eligible for the routine varicella vaccination | Study of effect on cost effectiveness if vaccination accelerates the onset of asthma Current programme | Markov model | no | no | 20 years | Single cohort; hypothetical | Change in costs | Societal | 3% costs  Outcomes not discounted |
| Esmaeeli 2017^58^ | Iran | Children 12 and 15 months old | 1 dose at 12 months  2 doses: 1 at 12 months the second at 15 months  No vaccination | Deterministic decision tree | no | no | Not reported | Single cohort; hypothetical | Cost-utility  (Cost per DALY) | Societal | none |
| Figueira 2003^34^ | United States | Refugees 1 to 20 years of age recently arrived in the US | Vaccinating those found susceptible by serotesting  1 dose under 13;  2 doses 13+ Presumptive vaccination | Single equation to identify the antibody prevalence above which it is less costly to test for antibody rather than vaccinate right away | no | no | Method abstracts from time. | Real: 637 refugee children | Cost minimising prevalence of antibody. | Healthcare | none |
| Gayman 1998^45^ | United States  Hospital in NC | 1. All employees  2. Employees caring for patients  3. Employees in high risk situations | 2 doses  1. Vaccinate without testing.  2. Test then vaccinate susceptibles  Test without vaccinating. | Adaptation of published Markov model | no | no | Not reported | Single cohort; hypothetical  10,000 | Cost savings and cost effectiveness ratios | Employer | none |
| Getsios 2002^70^ | Canada | Children 1 and 12 years old | 1. Universal vaccination at 1 year.  2. 1&catcup programme vaccinating susceptible 12 year olds  1 dose  No vaccination | Simulation model | no | no | 70 years | A series of birth cohorts | Change in cost and cost effectiveness  ratios | Healthcare and societal | 3% costs and benefits |
| Gialloreti 2005^71^ | Italy, Lazio | Infants and 11 year old susceptibles | 1 dose  No vaccination | Comparative statics | no | yes | 5 years | Numbers eligible for vaccination in one year | Annual cost savings | Healthcare | none |
| Ginsberg 2004^72^ | Israel | Susceptible Children 12 months old | 1 dose  No vaccination | Spreadsheet model | yes | no | Not reported | Birth cohort | Benefit:cost ratio | Healthcare and societal | 3% costs and benefits |
| Glantz 1998^38^ | United States | Women postpartum | Dosage not reported. Screening and vaccination of susceptibles  No screening or vaccination | Markov model | no | no | 55 years | Single cohort; hypothetical | ICER  Cost per event prevented. | Healthcare | 3% costs only |
| Goh 2016^39^ | Singapore | Army recruits | 1 dose Vaccination of all susceptibles  Vaccination of high risk susceptibles | Before-and-after study | no | no | 6 years: 3 before and 3 after | Six years of recruits | Change in costs | Employer | 1.237% costs only |
| Gray 1997^46^ | UK Hospitals | Hospital staff | V HV HSV SV  V: vaccinate  H: history of varicella  S: serotest  Sequence: eg  HSV: H then apply S to H- then vaccinate the S-.  2 doses  No vaccination | Decision tree | no | no | 10 years | Single cohort; hypothetical | Change in cost; ICER  Cost per case averted | Employer | 6%  costs only |
| Hammer-  schmidt 2003^94^ | Germany | Children 12-18 months old | 1 dose  No vaccination | Age-structured decision analytic model: Economic Varicella Vaccination Tool for Analysis (EVITA) | yes | no | 30 years | Single; hypothetical  birth cohort | Benefit:cost ratio | Payer and societal | 5%  costs only |
| Hanslik 2003^27^ | France | Adults 15-45 | HV  SV  H – varicella history  S – serotest  V - vaccination  2 doses  No vaccination | Decision tree | no | no | Lifetime | Single; hypothetical, though started at different ages from 15 to 45 | Change in costs  ICER: cost per event averted | Healthcare payer  Societal | 3% costs and benefits |
| Heininger 2021^51^ | Switzerland | Children 9 months to 24 months | Two doses  A MMRV 9 and 12 months  B MMRV 12 and 19 months  C MMRV 12 months/varicella vaccine at 24 months  Current practice (10% childhood coverage)  Catch up in susceptible 11-40 years olds | Age-structured, deterministic,  dynamic transmission model | yes | yes | 50 years | Hypothetical; successive birth cohorts | Cost per QALY  ICER | Healthcare and societal | 3% costs and benefits |
| Holl 2015^63^ | UK | Children aged 1, 3 and 12 | Universal vaccination at 1 and 3 years old. Catch up at 12.  2 doses  No vaccination | Age-structured dynamic transmission  model | yes | yes | Lifetime | Not clear. | ICER CUA  Cost per QALY | Healthcare | 3.5% costs and effects. |
| Howell 2000^41^ | United States | Army recruits | (a) serotesting at processing station followed by vaccination of susceptibles at initial entry training (IET)  (b) both at IET  (c) vaccinate all at IET  2 doses  No vaccination | Decision analytic probability model | no | no | Eight weeks (basic training period) | 100,000 annual recruits | ICER  Incremental cost per case prevented | Employer | 5% costs only |
| Hsu 2003^95^ | Taiwan | Healthy children at 15 months | 1 dose  No vaccination | Markov decision model | yes | no | Until cohort reaches 20 years old. | Single hypothetical  Birth cohort of 300,000. | Benefit:cost ratio  Net present value | Healthcare and societal  Parents’ willingness to pay. | 5% costs and benefits |
| Huse 1994^73^ | United States | Children at 15 months | 1 dose  No vaccination | Decision analytic model | no | no | 25 years | Single; hypothetical  100,000 | Change in costs | Healthcare and societal | 5% costs only |
| Kitai 1993^29^ | Canada  Hospital for Sick Children Toronto | Children eligible for liver or kidney transplant who have tested susceptible to varicella | 1 dose  No vaccination | Decision tree | no | no | 11.4 months | Single true/hypothetical.  44 varicella susceptible children | Cost savings per child to health service and to parents | Healthcare and societal | Not relevant: short time horizon |
| Lenne 2006^80^ | Spain | Children 1-11 | (a) Universal vaccination at 1 year old  (b) (a) + Catch up of 2-11 year olds  1 dose  No vaccination | Dynamic transmission model | yes | no | 50 years | Single, hypothetical.  Birth cohort 440,000 | Cost changes, benefit:cost ratio, cost effectiveness: cost per life year saved | Healthcare, health system, societal | 3% costs only |
| Lieu 1994^74^ | United States | Children under 6 | 1 dose  No vaccination | Decision analysis | no | no | 30 years | Single hypothetical birth cohort of 4m | Cost changes,  benefit:cost ratio.  Cost effectiveness:  Cost per event prevented | Healthcare and societal | 5% costs;  Results illustrated with and without 5% applied to health effects. |
| Lieu 1995^25^ | United States | Schoolchildren 6-12  Adolescents 13-17 | Children  (a) SV (b) V  Adolescents  (c) SV (d) V (e) VS (to test need for a second dose)  V: vaccinate  H: history of varicella  S: serotest  Sequence: eg  HSV: H then apply S to H- then vaccinate the S-.  1 dose except for a proportion of (e) 2 doses  No vaccination | Decision analysis | no | no | 30 years | Two single hypothetical cohorts of 10,000, one of children, one of adolescents | Cost changes.  Cost effectiveness:  Cost per case prevented. | Healthcare and societal | Costs 5%  Health outcomes not discounted |
| Littlewood 2008^83^ | France | Those eligible for MMR | MMRV  MMR | Dynamic transmission model | no | yes | 30 years | Not reported | Cost changes;  Cost per QALY. | Healthcare; QALYs | Not reported |
| Littlewood 2009^84^ | France | Those eligible for MMR | (a) 80% of MMR over 5 years  2 doses  (b) 100% of MMR over 2 years plus catch up programme  2 doses  1 in catch up MMR | Age-structured dynamic transmission model | no | no | 30 years | Not reported | Cost changes;  Cost per QALY. | Healthcare and societal | Not reported |
| Marijam 2022^61^ | Russia | Children  12 months, 15 months and 6 years. | Two doses.  A 12 and 15 months  B 12 months and 6 years  No vaccination | Markov model | no | no | 10 years | Hypothetical;  Single birth cohort. | Cost per QALY  Cost per case averted | Healthcare and societal | 3.5% costs and benefits |
| Melegaro  2018^54^ | Italy | 1. 15 months  2. 5-6 years old | 2 doses  First dose at 15 months of age, second dose at 5–6 years old  No vaccination | Epidemiological model | no | yes | 25/50/85 years | Single; Italian National Immunisation Plan (NIP) | CUA: Cost/QALY | Health payer | 3% costs and benefits |
| Merrett  2007^35^ | Canada | Newly arrived adult immigrants and refugees | 1 dose  No vaccination | Markov decision tree | no | no | 20 years | Single; hypothetical  Cohort aged 30 years | CUA: QALY gained | Societal | 3% costs and benefits |
| Nettleman  1997^47^ | United States | Healthcare workers in the University of Iowa hospital | 2 doses  No vaccination | Markov decision tree | no | no | 1 year | Single; hypothetical  cohort | Net cost savings | Healthcare | 5% costs |
| Olson  2001^30^ | United States | Susceptible children eligible for kidney transplant | Pre-treatment vaccination  2 dose  Usual care | Decision tree | no | no | 5 years | Single;  Children's Hospitals,  at the University of Michigan | CEA: Cost per case prevented | Healthcare | 5% costs |
| O’Neill  2003^48^ | UK | Paediatric staff | 2 doses  No vaccination | Survey | no | no | 5 years | Single;  22 UK paediatric hospital units | Not clearly specified | Assumed employer but not clearly specified | Not clearly specified |
| Paternina-Caicedo  2013^52^ | Colombia | Children 12-15 months | 1) 1 dose at 12 months  2) 1) & a second dose at 15 months  No vaccination | Decision tree | no | no | 30 years | Multiple;  1) vaccinated children under 2 years old  2) one set of cohort: no varicella vaccine was used  3) in the second set, 30 consecutive cohorts of newborns  (850 thousands) received one dose of varicella  3) 30 cohorts of newborns received the two dose scheme | CEA: Life year gained Reduction of consultations | Health care system perspective | 3% for costs |
| Patrikar 2022^42^ | India | Recruits to armed forces | Two doses  No vaccination | Before and after study. | no | no | 2 years after vs 9 years before | Natural: annual recruits to armed forces | Cost per case averted and cost per man day saved; cost per QALY | Employer;  Also patient | n/a |
| Pawaskar 2021^62^ | Norway | Children at 15, 18 months, 7,11 years. | Two doses.  No vaccination  First dose 15 months  Second dose  Strategy  AB 7 years  CD 11 years  EF 18 months  AE BF C D  Va Vl Va Vl  Va Vl PQ PT  Va: Varivax  Vl : Varilrix  PQ: ProQuad  PT: Priorix-Tetra | Dynamic transmission model | yes | yes | 50 years | Hypothetical  Successive birth cohorts | Cost savings  QALYs gained  Cost per QALY ICER | Healthcare and societal | 3% costs and benefits |
| Peña Blasco 2017^75^ | Spain  Aragón | Children 1 year and 3 years old | 2 doses  No formal programme | Retrospective hypothetical treatment over a time period | no | no | 11 years | True cohort: Eligible children over 11 years | Benefit:cost ratio | Societal | 3% costs only |
| Pinot de Moira  2006^37^ | UK | Primigravidae UK or Bangladesh born | 2 doses  No vaccination | Decision tree | no | no | 30 years | Multiple cohorts of UK-born  and Bangladesh-born primigravidae aged 15–44 years. | CEA | Health care provider | 3.5% |
| Preblud  1985^96^ | United States | 15 months children | Dose not reported  No vaccination | Not clearly specified | no | no | 30 years | Multiple;  1) Hypothetical birth cohort of 3.5 million persons at 15 months of age. | CEA  Benefit:cost ratio  Cost savings | Societal | Not specified |
| Rafferty 2021^55^ | Canada, Alberta | Children 12 months, 18 months and 4-6 years | Two doses  A: 12 and 18 months  B: 12 months and 4-6 years  No vaccination | Agent-based model | yes | yes | 75 years | Hypothetical  Successive birth cohorts | Cost per QALY ICER | Healthcare and societal | 1.5% costs and benefits |
| Sauboin 2012^85^ | Italy | Children receiving MMR | MMRV  2 doses  MMR | Age-structured dynamic model | no | yes | Lifetime of cohort | Single hypothetical. | Cost utility:  cost per QALY | Healthcare | Not reported |
| Scuffham  1999^76^ | New Zealand | 15 months children | Dose not reported  No vaccination | Decision tree | no | no | 30 years | Single an annual cohort of 57,200, 15-month old children | CEA  Cost per event prevented  Benefit:cost ratio | Health care purchaser | 5% |
| Scuffham  2000^77^ | Australia | 12 months old and 12 years old | 1. All infants  2. Susceptible adolescents  3. 1&2 for 11 years  1 dose  No vaccination | Decision tree | no | no | 30 years | Single:  annual birth cohorts of infants (12-months old) and adolescents (12 years old). | CEA  Cost per event prevented | health-care perspective | 5% |
| Smith  1998^36^ | United States | Women 15-49 postpartum | Postpartum vaccination of susceptibles identified prepartum  2 doses  No vaccination | Decision tree | no | no | 20 years | Single: a hypothetical cohort of 4 million women (15-49 years old) | CEA:  Cost per event prevented | Health care payer perspective/ societal | 5% |
| Smith 2000^28^ | United States | 20-29-year-olds or 30+ | 1. Serotesting and vaccination of susceptibles  2. Vaccinate all  2 doses  No vaccination | Decision tree | no | no | 100 years | Hypothetical cohorts of adults 20-29 and 30 and over | Cost utility  Cost per QALY | Societal perspective and health care payer | 3% costs and benefits |
| Thiry  2004^26^ | Italy | 11 year olds | 1. Vaccinate all  2. Vaccinate susceptibles after testing  3. Private vaccination  1 dose  No vaccination | Decision tree | no | no | Lifetime | Multiple: Identical hypothetic cohorts of 20- to 29-year-old or 30-year old and older patients | CEA  Cost per event prevented | Societal and third-party payer | 3% |
| Thompson 2012^86^ | UK | Children 12 months old | Dose not reported  No vaccination | Age-structured (susceptible, infectious,  recovered) dynamic transmission model | yes | no | 75 years | Single hypothetical | Cost utility:  cost per QALY | Healthcare | 3.5% for costs and benefits |
| Tseng  2005^97^ | Taiwan | 300,000 infants | Dose not reported  No vaccination | Cohort based model | no | no | 30 years | Single : A new 300,000 hypothetical birth cohort | CEA:  Cost per event prevented | Societal perspective and health care payer | none |
| Valentim 2008^103^ | Brazil | Children 12 months old | 1 dose  Some vaccination of vulnerable groups; some private vaccination | Decision analysis incorporating susceptible-infectious-recovered- vaccinated model. | yes | no | 30 years | Single hypothetical | Cost changes  Cost effectiveness:  Cost per event prevented. | Healthcare and societal | 6% costs  3% outcomes |
| Valentim 2009^102^ | Brazil | Not reported | Study to rework previous estimates of ICER in childhood vaccination using fuller costings.  Comparator not reported | Not reported | Not reported | Not reported | Not reported | Not reported | Impact on cost per life year saved | Health care and societal | Not reported |
| Van Hoek 2012^56^ | UK | Those eligible for childhood vaccination; the elderly 65+ | 1. Childhood varicella 2 doses  2. Elderly herpes zoster 1 dose  3. 1&2  No vaccination | Dynamic transmission model | yes | yes | 100 years | Not reported | CUA:  Cost per QALY in relation to a threshold. | Healthcare  NHS | 3.5% cost and benefits |
| Van Lier 2015^87^ | Netherlands | Children 12 months and 4 years | 2 doses, one at 12 months second at age 4 No vaccination | Age structured transmission model | Not reported | yes | 180 years | Successive birth cohorts | CUA  Cost per QALY | Societal | 4% costs.  1.5% QALYs |
| Wang 2021^57^ | China,  Jiangsu | Children 12 and 15 months | One or two doses, universal or selected by serotesting.  Strategy A B C D  Doses 1 1 2 2  Universal U or selective S  U S U S  No vaccination | Markov model | no | no | 60 years | Hypothetical  Single birth cohort | Cost per QALY ICER | Societal | 3% costs and benefits |
| Wolff 2021^88^ | Sweden | Children 12 and 18 months | Two doses.  No vaccination | Age-structured dynamic Markov transmission model | yes | yes | 85 years | Hypothetical  Successive cohorts of 12 month olds | Cost per QALY ICER | Societal | 3% costs and benefits |
| Wolfson 2019^53^ | Turkey | Children 12, 18 months and 6 years old | 1 dose at 12 months (a)  Repeat dose at 18 months (b) or 6 years (c).  No vaccination | Dynamic transmission model | yes | yes | 100 years | Single hypothetical | Cost changes.  Cost utility analysis  Cost per QALY | Health care and societal | 3% costs  and outcomes |
| Zhou 2005^78^ | United States | Children 12-15 months old | 1 dose  No vaccination | Decision tree | no | no | Lifetime of birth cohort | Single cohort; hypothetical 2001 US birth cohort | Net present value and benefit:cost ratio | Healthcare and societal | 3% costs and benefits |
| Zhou 2014^79^ | United States | Those eligible for the routine childhood immunisation programme | 2 doses  No vaccination | Decision tree | no | no | Lifetime of birth cohort | Single cohort; hypothetical 2009 US birth cohort | Net present value and benefit:cost ratio | Healthcare and societal | 3% costs and benefits |

**Table 2 Results: Impact on cases and cost: Results of economic evaluation by perspective – healthcare and societal (Costs are presented in 2021 £ sterling unless otherwise stated)**

| Study | Impact | | Cost-Effectiveness/Utility/Benefit Results | | | Quality markings | |
| --- | --- | --- | --- | --- | --- | --- | --- |
| Author,  date | Cases, hospital  admissions, mortality | Cost | Healthcare | Societal | Threshold analysis | CHEERS | CHEC |
| Akpo 2020^59^ | Reduction in incidence (MMRV/MMRV) 95.6%,  (V-MMRV) 91.0%.  HZ 98% regardless of boosting  MMRV-varicella vaccination along with measles, mumps, rubella vaccination  V-MMRV – varicella vaccination for the first dose, MMRV for the second dose | Direct +20.4%  Indirect  -30.1%  Societal  -0.07% | Cost/QALY  V-MMRV £5665 | Cost/QALY  V-MMRV  dominates | Both healthcare and societal meet £20,000 threshold | 23 | 16 |
| Azzari  2020^81^ | Reduction in  Cases 52%-66%  Hospital admissions 57%-71%  Deaths 20%-30%  QALYs/person 0.01 | Health care -60%  Societal -58% | Vaccination dominates | Vaccination dominates | Vaccination dominates | 22 | 15 |
| Banz 2003^49^ | Strategies: Children (1), Adolescents (2) Both (1&2)  Reduction from no vaccination %  1 2 1&2  Cases/  Complics 82.7 5.0 83.5  Deaths 81.8 9.2 81.8 | Annual net savings  Societal  £m  1 2 1&2  61.6 9.8 63.6 | Benefit-Cost ratio vs no vaccination  1 2 1&2  Payer 1.75 1.13 1.70 | Benefit-Cost ratio vs no vaccination  1 2 1&2  4.12 8.44 4.10 | None | 21 | 12 |
| Banz 2009^90^ | Strategies: Children (1)  (2) plus adolescent catch up  Reduction from current adolescent vaccination %  1 2  Cases 75 78  Complics 73 76  Hospital  admissions 65 70  Deaths 100 100 | Change in costs compared with current adolescent vaccination %  1 2  Payer 85 106  Societal -10 -9 | Benefit-Cost ratio vs current policy    Strategy 1 2  Payer 0.30 0.27 | Benefit-Cost ratio vs current policy  Strategy 1 2  Societal 1.29 1.22 | None | 21 | 13 |
| Baracco 2015^43^ | Risk of varicella annual reduction vs no policy %  H, S, HS -  HSV 7.7  HV 38.7  SV 40.0  V 56.6  V: vaccinate  H: history of varicella  S: serotest  Sequence: eg  HSV: H then apply S to H- then vaccinate the S-. | Not reported | Cost per case prevented  £k  HS comparator  HSV 40.3  HV 65.2  SV 75.3  V 341.0 | None | None | 20 | 16 |
| Beutels 1996^67^ | Annual varicella-related health events: % reduction by strategy: children (1) 12 year olds (2) both  1 2 1&2  Infections 57 37 55  Hospital  admissions 29 36 31  Deaths 20 35 25  Life years  lost 38 37 37 | Costs compared with no vaccination per cohort £m  1 2 1&2  Direct 10 - 5 5  Indirect -206 -21 -240  Total -197 -26 -235 | Direct benefit:cost ratio  1 2 1&2  0.82 1.94 0.92 | Total benefit:cost ratio  1 2 1&2  4.6 6.02 4.72  Direct costs per  infection prevented £  25.1 -163.7 11.7  death prevented £m  2.4 1.1 0.6  life year gained £k  28.2 62.6 11.6 | None | 18 | 16 |
| Bilcke 2013^82^ | A two dose regime at 1 and 11: 97% reduction in cases which persists. | Primary varicella: savings rise steadily for 100 years.  Breakthrough varicella: rise for 60 years then tail off.  Break even by year 60.  Vaccination at age 1 and 11 with exogenous boosting. | Options  (a) 2 doses with exogenous boosting  (b) 2 dose + 1 dose zoster and favourable model assumptions  (c) 2 dose no boosting  Number of years until cost per QALY meets passmark of €35,000:  (a) 100 years  (b) 40 years  (c) qualifies from inception | none | See healthcare box | 22 | 16 |
| Blas 2017^98^ | 34m cases and 557 deaths averted over 10 years.  1 dose  A: at 12m B : at 18m  2 dose  C: A+at 18m; D: A+at 4years | All options had lower costs. | All options cost saving.  D, followed by C, A, and B, | Not applicable | None | 18 | 11 |
| Boccalini 2020^32^ | Avoided cases per 1000  Comparator 3.5382  (a) serotesting 3.6623  (b) presumptive  vaccination 3.5566 | Per 1000 children £  Comparator 23,945  (a) serotesting 21,391  (b) presumptive 25,452 | ICER per case prevented  Serotesting dominates  presumptive £394.92 | Not applicable | None | 19 | 11 |
| Bonanni 2008^91^ | Reduction by strategy %  1 2 3  Cases 81.3 10.3 82.3  Consultations 74.8 7.8 76.1  Complications 81.7 12.1 82.4  Hospital  admissions 80.8 17.1 82.1  Deaths 75.0 50.0 75.0  1 – children 1-2 yrs  2 – adolescents 13 yrs  3 – 1 plus susceptible adolescents | Change in cost by strategy %  1 2 3  Direct 38 23 44  Indirect -73 -18 -74  Total -52 -10 -52 | Benefit-cost ratio by strategy    1 2 3  0.67 0.36 0.64 | Benefit-cost ratio by strategy  1 2 3  3.47 2.60 3.33 |  | 21 | 12 |
| Brisson 2002^92^ | Reduction in varicella by strategy %  1 2 3  Cases 97 87 12  Consultations 96 81 17  Hospital  admissions 71 85 15  Deaths 52 63 12  Increase in zoster strategy 1 (infants)  %  Consultations 14  Hospital admissions 21  Deaths 22  1 – children at 1 year  2 –1 yr & 5-11 yrs catch-up  3 – preteen 12 yrs | Change in cost by strategy %  1 2 3  Direct 40 54 5  Indirect -75 -87 -11  Total -60 -69 -9 | Benefit-cost ratio by strategy  1 2 3  0.61 0.60 0.73* * incl zoster 0.16  Discounted cost per life year gained £k  1 2 3 3*  41 47 17 109 | Benefit-cost ratio by strategy  1 2 3  5.24 4.90 4.40 | Only strategy 3 satisfies the passmark of £23k per life year | 21 | 15 |
| Brisson Edmunds 2003^64^ | Reduction in number of cases/events over 80 years compared with no vaccination. k - thousands except where specified  Strategy 1 2 3  Varicella  Cases m 14.6 15.7 1.9  Consultations 8.0 8.7 1.5  Hospital  admissions 46.9 51.9 10.5  Deaths (nos) 22 199 128  Life years 14.2 17.4 7.4  QALYs 80.2 88.5 18.6  Strategy 1 2 3  Zoster  Cases m -0.6 -0.7 -0.1 Consultations -1.1 -1.3 -0.1 Hospital  admissions -15.5 -18.4 -0.1  Deaths (nos) -444 -526 -41  Life years -4.1 -4.9 -0.4  QALYs -134.6 -145.7 -11.0  1 – children at 1 yr  2–1&catch-up in 2-11 year olds in first year  3 – adolescent: 11 year old susceptibles | 80-year discounted costs compared with no vaccination £m  Strategy 1 2 3  Direct 562 811 195  Indirect -165 -404 -437  Total 396 407 -241 | Strategies 1-3 compared with no programme  1 Cost + QALY -  2 Cost + QALY -  3 Cost/QALY £26,000 | Strategies 1-3 compared with no programme  1 Cost +  2 Cost +  3 Cost - | Not reported | 22 | 16 |
| Burnham 1998^39^ | Reduction in cases and hospital admissions 86% | Reduction in Annual cost for 1400 cadets 72% | Reduction in cost pa:  £93.9k | none | none | 8 | 12 |
| Chacon-Cruz 2022^60^ | Cases averted over 20 years  Strategy  A one dose 20.6m  B-D two doses 23.0m  A: one dose at one year  B: two doses at 1 and 6  C: one dose at 1, one dose MMRV at 6  D: two doses MMRV, at 1 and 6 | Cost saved over 20 years  Strategy  A £37.7m  B £34.4m | Cost per case prevented and cost per life year gained (LYG)  Strategy case LYG  A d  B d d  C £26 £1093  D £80 £8027  d: dominates | As for healthcare | Cost per LYG against annual GDP per head.  Strategy C meets threshold  Strategy D does not. | 17 | 15 |
| Chodick 2005^44^ | Reduction in cases over 20 years: eligible cohort  Strategy  HSV 43  SV 53  V 54  V: vaccinate  H: history of varicella  S: serotest  Sequence: eg  HSV: H then apply S to H- then vaccinate the S-. | Cost of testing and vaccination over 20 years: eligible cohort  Strategy £k  HSV 574.6  SV 1135.1  V 4248.8  Cost of treatment and furloughing not reported. | Incremental cost per case prevented  Strategy £k  HSV 22.8  SV 198.4 V 9837.0  Cost savings from reduced precautions not included. | none | none | 19 | 15 |
| Chui 2014^31^ | Reduction in QALY loss per patient vaccinated: 62% | Reduction in hospital cost per patient vaccinated: 56% | Vaccination dominates: lower cost and lower QALY loss. | none | Intervention dominates. | 21 | 16 |
| Coudeville 1999^101^ | Reduction in cases and complications %  Cases 42 Complications 89  Reduction in work absences 87% | Reduction in discounted present value over 30 years of direct medical costs: 57% | Savings in discounted present value over 30 years of direct medical costs: £347m | none | none | 20 | 15 |
| Coudeville 2003^99^ | Decrease in cases in a 50 year time frame: 85% | Reduction of 4% in direct medical costs and 10% in social security costs | Reduction of 4% in direct medical costs and 10% in social security costs | none | none | 17 | 14 |
| Coudeville 2004^89^ | Average annual percentage reductions over a 50 year period: vs none (1) and as increments over (1) for (2) and (3))  Strategy 1 2 3  Cases 82 34 45  Hospital  Admissions 68 27 37  Deaths 57 12 19 | Average annual percentage change in discounted cost over a 50 year period: vs none (1) and as increments over (1) for (2) and (3)) %  Strategy 1 2 3  direct -16 5 2  indirect -49 -19 -26  Societal -40 -11 -15 | Heading as previous cell  Strategy 1 2 3  -16 5 2 | Heading as previous cell  Strategy 1 2 3  -40 -11 -15 | none | 21 | 15 |
| Coudeville 2005^100^ | Average annual reduction over a 50 year period:  (1) vs none and (2) vs (1) %  1 vs 0 2 vs 1  France  Cases 94 54  Complications 92 49  Deaths 82 50  Life-years lost 90 41  Germany  Cases 93 63  Complications 93 59  Deaths 90 -  Life-years lost 91 49 | Reduction in discounted costs over 50 years %  Infant strategy  societal public  sector  France 59.6 6.7  Germany 61.4 51.1  Increment from catchup  societal public  sector  France 8 5  Germany 11 -5 | Reduction in discounted costs over 50 years %  Infant strategy  France 6.7  Germany 51.1  Increment from catchup  France 5  Germany -5 | Reduction in discounted costs over 50 years %  Infant strategy  France 59.6  Germany 61.4  Increment from catchup  France 8  Germany 11 | none | 21 | 15 |
| Damm 2015^50^ | Effect of discontinuing current universal vaccination programme  QALY impact by assumptions about exogenous boosting  Boosting with without  gain loss | Effect of discontinuing current universal vaccination programme  Cost impact by assumptions about exogenous boosting  Boosting with without  lower lower | none | Effect of discontinuing current universal vaccination programme  Boosting  QALY Cost  With + -  Without - - | none | 17 | 15 |
| De Boer 2013^68^ | For birth cohort of 180,000  Cases averted 105,091  QALYS gained 301.1 | For birth cohort of 180,000  Cost saving  £13.4m direct and indirect costs | €40,582 (£39,770)/QALY | €2844 (£2787)/QALY | Cf Netherlands passmark €20,000/QALY | 17 | 14 |
| De la Hoz 2011^65^ | Reductions in birth cohort  Consultations 9,415,444  Hospital  admissions 17,576  Deaths 1,144 | Reductions in costs  60% | Per life year gained  £2173 | none | Satisfies WHO passmark of GDP per head. | 20 | 15 |
| De Valliere 2011^33^ | Cases  Before 7  After 0 | Percentage change in cost per person  167% | Change in cost only | Cost per case prevented implicit but not stated. | none | 15 | 12 |
| Diez Domingo 1999^93^ | Cases/events saved in birth cohort followed for 20 years  Children Adults  Cases 652,986 n/a  Consultations 423,837 61,902  Outpatients 28,523 4,127  Inpatients  Uncomplicated 857 124  Complicated 224 33  Days off work Parents Adults  657,542 143,341 | Percentage change in costs  Medical 73  Indirect -86  Total -32 | Cost:benefit ratio (sic)  (actually the benefit:cost ratio)  0.54:1*  *Reduction in medical costs divided by costs of vaccination | Cost:benefit ratio (sic)  (actually the benefit:cost ratio)  1.61:1 | The benefit cost ratio of 1.61 for the societal perspective indicates good value for money. | 21 | 16 |
| Dimitrova 2018^66^ | Percentage reduction in cases  2 dose strategy 88% | Percentage change in costs  2 dose strategy 51% | ICER €3600 (£4140) | none | Satisfies WHO passmark of GDP per person of €18,973 | 17 | 10 |
| Ditkowski 2016^69^ | Change in asthma events if vaccination brings forward the onset of asthma by 9 years: birth cohort of 3,957,577  Morbidity 9,980  Deaths 127 | Percentage reduction in cost saving of programme if onset of asthma brought forward by 9 years  36% | Not reported | 36% reduction in cost savings | Net cost savings remain positive if vaccination accelerates the onset of asthma by 9 years | 22 | 16 |
| Esmaeeli 2017^58^ | Reported percentage reduction in events lost are all equal to the reported vaccine efficacy ie one dose 85%, two doses 95%. | Percentage change in cost  I dose 101%  2 doses 271% | Not reported | Cost per DALY vs no programme  1 dose $17,280  (£13,478)  2 doses $41,531  (£32,394) | Not cost effective on the three times GDP per head benchmark:  £14,292 for Iran | 22 | 14 |
| Figueira 2003^34^ | Not reported | Less costly to test if prevalence exceeds p*  $p*=\frac{S}{V-[(1-PV)DAEC]}$  p* – prevalence of antibody  S – screening cost  V – vaccination cost  PV – PPV of ELISA test  D – mean cost of disease  A – attack rate in susceptibles  E – vaccine efficacy  C – vaccine compliance | The critical value of antibody prevalence (*p**) was 34% in children < 13 years old (who required only one dose), and 17% in children 13 years old and over (two doses) | none | Cost effective  (cost minimising) to vaccinate children 1-4 without testing; to test other ages. | 20 | 15 |
| Gayman 1998^45^ | Reduction in infections among employees  Both strategies 0.35%  Reduction in patient exposures per employee  All employees 6.7%  Employees in direct care or high risk environments 15.0% | Percentage cost change  Employee S&V V  roles  All -31 77  Direct care -40 42  High risk -48 11  V – vaccinate all  S&V – screen then vaccinate | Cost per event prevented £k  Employee Infection Employee  roles S&V V  All 16 41  Direct care 35 45  High risk 22 50  Patient exposure  Employee  Roles S&V V  All 0.8 2.2  Direct care 0.5 1.1  High risk 0.5 1.2 | none | none | 15 | 11 |
| Getsios 2002^70^ | Reduction in events (cohort of 100,000) vs no programme %  Strategy 1 1&catchup  Cases 63 66  Hospital  Admissions 62 75  Deaths 48 69  1 - universal vaccination of 12 month olds | Reduction in cost (cohort of 100,000) vs no programme %  Strategy 1 1&catchup  Healthcare 42 45  Non-  Healthcare -73 -78  Total -29 -31  - | Eleven cohorts of vaccinated children  Cost per case avoided £  Strategy  1 39*  1&catchup 59^§^  Cost per .life year gained £k  1 78*  1&catchup 39^§^  *Compared with no vaccination  §Increment over 1 | Vaccination at 12 months dominates no vaccination.  Vaccination at 12 months plus catchup programme at age 12 dominates vaccination at 12 months. | none | 21 | 17 |
| Gialloreti 2005^71^ | Reduction in varicella cases over a five year period: 79% | Reduction in varicella costs over a five year period: 38% | none | As in cost cell. | Favourable but sensitive to an increase in herpes zoster | 20 | 12 |
| Ginsberg 2004^72^ | Reductions %  Cases 92  Life years lost 92  Deaths 92 | Reduction in costs %  Healthcare 27  Work, travel etc 93  Total societal 93 | Benefit:cost ratio  1.63 | Benefit:cost ratio  19.33 | none | 19 | 17 |
| Glantz 1998^38^ | Lifetime reduction in events %  Cases 35  Life years lost 0.5  Deaths 16  (from pneumonia) | Reduction in varicella-related discounted cost per person treated over 55 years  25% | Screening & vaccination option dominates |  | none | 21 | 16 |
| Goh 2016^40^ | Reduction in events in the three years after adoption of the universal option vs the three years before %  Cases 60  Outbreaks 93  Days lost 82 | Change in costs in the three years after adoption of the universal option vs the three years before %  Days lost -56  Outbreak  management -92  Outpatient and  medication -61  Vaccination 734  Side effects 896    Total -12 | Cost saving of 12% | none |  | 20 | 16 |
| Gray 1997^46^ | Reduction in staff index events %  V HV HSV SV  90 85 85 90  V: vaccinate  H: history of varicella  S: serotest  Sequence: eg  HSV: H then apply S to H- then vaccinate the S-. | Change in ten year discounted costs per average hospital %  V HV HSV SV  224 39 -2 63 | Cost per incident averted vs baseline option £k  V HV HSV SV  67 12 0 18 | none | none | 22 | 17 |
| Hammer-schmidt 2003^94^ | Not reported | Not reported | Benefit:cost ratio  (cost savings divided by cost of vaccination)  Payer 1.75  0.74*  *if parental absence not covered by health insurer | Benefit:cost ratio  Societal 4.12 | none | n/a | n/a |
| Hanslik 2003^27^ | Lifetime discounted events etc avoided per 100,000 15 year olds  Strategy HV SV  Cases 6780 6712  Hospital  admissions 99 98  Deaths 1.58 1.57  Life years saved 41.2 40.8  V - vaccinate  H - history of varicella  S - serotest  Sequence: eg  HV: H then vaccinate the H-. | Change in lifetime discounted cost per 15 year old %  HV SV  Direct 200 96  All 15 -14 | Cost per case avoided 15 year old £  HV SV  43,964 389  Cost per death avoided £  HV SV  8.7m 63,916 | Cost per case avoided 15 year old £  HV SV  43,384 -ve  Cost per death avoided £  HV SV  8.6m -ve |  | 20 | 17 |
| Heininger 2021^51^ | Changes  (results for strategies A-C not materially different)  Cases - 88%  Hospital  admissions - 62%  Deaths - 75%  QALYs lost - 20%  A - MMRV 9 and 12 months  B - MMRV 12 and 19 months  C- MMRV 12 months/varicella vaccine at 24 months | Change in cost  (results for strategies A-C not materially different)  Healthcare - 80%  Societal - 84% | Cost per QALY vs current situation    Strategy  A £18,542  B £18,204  C £20,944 | Cost per QALY vs current situation    Strategy  A £15,219  B £14,814  C £17,472 | All ICERs met threshold of annual GDP per head. | 23 | 16 |
| Holl 2015^63^ | Cases avoided by years following inception of programme %  Years 5 15  57.7 94.8 | Costs saved by years following inception of programme £m  Years 5 15  Outpatient 25.2 93.8  Hospital  admission 5.9 19.8 | Cost per QALY by years following inception of programme (95% CLs) £  Years 5 15  6794 7267  (-418:14940) (381:14902) | none | none | 21 | 12 |
| Howell 2000^41^ | Events avoided vs no vaccination %  Strategy (a) (b) (c)  Cases 11 19 25  Complications 39 69 90  Deaths 30 50 60  (a) serotesting at processing station followed by vaccination of susceptibles at initial entry training (IET)  (b) both at IET  (c) vaccinate all at IET | Cost per 100,000 recruits £m  Strategy - (a) (b) (c)  Vacc - 3.6 3.1 20.7  hospital/  training 0.2 0.18 0.17 0.16  Total 0.2 3.8 3.2 20.9  Change vs no vaccination %  (a) (b) (c)  1,811 1,522 10,333 | Cost per case prevented: increment of strategy on the x axis over the one on the y axis £k  (b) (a) none  none  (a) 0.9  (b) -1.2 0.4  (c) 8.8 3.4 2.3  For example, the incremental cost per case prevented by strategy (b) over none is £0.4k | none | none | 17 | 16 |
| Hsu 2003^95^ | Not reported | Change in cost %  Healthcare 138  Societal -48 | Benefit:cost ratio 0.34 | Benefit:cost ratio 2.06  NPV of willingness to pay £-13.6m discounted total up to age 20 for birth cohort of 300,000. | none | 21 | 16 |
| Huse 1994^73^ | Reduction of events %  Cases 95  Consultations 93  Medication 95  Hospital admissions 95 | Change in costs to age 25 %  Healthcare 177  Work loss -95  Total -95 | Change in costs to age 25: 177% | Change in costs to age 25: -95% | none | 22 | 16 |
| Kitai 1993^29^ | Only reported in terms of costs. | Reduction in costs %  Healthcare 89  Parents 96  Total 89 | Cost savings per child  £3167 | Cost savings per child  Parents £339  Total £3508 | Threshold analysis only in sensitivity analysis. Threshold defined as positive cost savings | 19 | 16 |
| Lenne 2006^80^ | Reductions in events over a 50 year period compared with no vaccination %  (a) (b)  Cases 89 93  Complications 84 90  Hospital  admissions 80 84  Deaths 65 83  (a) Universal vaccination at 1 year old  (b) (a) + catch up of 2-11 year olds | Change in discounted costs over a 50 years period 440,000 birth cohort %  (a) (b)  Treatment -83 -91  Healthcare -4 -2    Indirect -66 -78  Societal -51 -60  Health system  Treatment -83 -91  Healthcare 9 12 | Change in discounted costs vs no vaccination %  (a (b)  Healthcare -4 -2  Health  System 9 12  Benefit:cost ratio  (a) (b)  Healthcare 1.05 1.02 Health  System 0.91 0.88  Cost per life year saved £  (a) (b)  Health  system 5376 17,971 | Percentage change in discounted costs vs no vaccination  (a) (b)  -51 -60  Benefit:cost ratio  (a) (b)  2.67 2.77  Cost per life year saved £  (a) (b)  Not 11,6761  reported | £5376 cost per life year saved in the healthcare system deemed cost effective. | 21 | 16 |
| Lieu 1994^74^ | Reduction in average annual events %  Cases 94  Major  sequelae 94  Long term  disability 95  Deaths 93 | Change in discounted costs: Annual average first 30 years of programme %  Medical 9  Work loss -89  Total -72 | Percentage change in discounted costs vs no vaccination: 9  Benefit:cost ratio 0.9 | Percentage change in discounted costs vs no vaccination: -72  Cost per  Case avoided  £5.38  Cost per major complication avoided £2112  Cost per life year saved £20,480  Outcomes discounted  Benefit:cost ratio 5.4 | none | 20 | 16 |
| Lieu 1995^25^ | Reduction in cases over 30 years %  Children (a) (b)  89 95  Adolescents  (c) (d) (e)  81 98 88  Children  (a) SV (b) V  Adolescents  (c) SV (d) V (e) VS (to test need for a second dose)  V: vaccinate  H: history of varicella  S: serotest  Sequence: eg  HSV: H then apply S to H- then vaccinate the S-. | Change in costs %  Children (a) (b)  Medical 308 334  Societal 0 -22  Adolescents (c) (d) (e)  Medical 478 1124 715  Societal 31 137 74 | Cost per case prevented £  Children (a) (b)  150 152  Adolescents  (c) (d) (e)  289 562 395 | Cost per case prevented £  Children (a) (b)  savings  Adolescents  (c) (d) (e)  105 388 232 | none | 20 | 16 |
| Littlewood 2008^83^ | Reduction in cases per million patient-years 61%  Unquantified reduction in complications and zoster after a slight blip. | Annual reduction in indirect costs: £1.8m | £14,575 per QALY | MMRV dominant over MMR. | none | 19 | 14 |
| Littlewood 2009^84^ | Reduction in cases per million patient-years %  (a) (b)  61 83  (a) 80% of MMR over 5 years  2 doses  (b) 100% of MMR over 2 years plus catch up programme  2 doses  1 in catch up | Reduction in annual inpatient and outpatient costs: 32% | Cost per QALY (CLs) £  (a) (b)  3347 3284  (-1:9927) (15:8916) | Both (a) and (b) dominant. | none | 19 | 14 |
| Marijam 2022^61^ | Changes vs no vaccination  Strategy A B  Cases -91% -73%  GP visits - 89% - 67%  Hospital  admissions - 96% - 95%  QALYs lost - 96% - 91%  A 12 and 15 months  B 12 months and 6 years | Change in costs  Strategy A B  GP visits - 89% - 65%  Hospital - 96% - 95%  admissions  Indirect costs - 91% - 72% | Cost per QALY and cost per case prevented  Strategy A B  Cost/QALY £k 50.3 50.9  Cost per case  Prevented £ 180 214 | Cost per QALY  Both A and B dominate no vaccination | All cost/QALY ICERs meet threshold of £60.9k | 23 | 15 |
| Melegaro  2018^54^ | Reduction in QALY losses vs no vaccination time horizon 25 years %  1. Model TI (Temporary immunity)  V H VH H+cu VH+cu  2 9 12 16 19  2. Model PI (progressive immunity)  V H VH H+cu VH+cu  - 11 11 17 18  V-varicella H-herpes zoster cu-catch up VH – V+H | Increase in cost vs no vaccination %  1. Model TI (Temporary immunity)  V H VH H+cu VH+cu  1 40 41 56 57  2. Model PI (progressive immunity)  V H VH H+cu VH+cu  2 35 36 48 50 | Vaccination dominates  VH+cu dominates all options except no vaccination and V in model T1 |  |  | 21 | 17 |
| Merrett  2007^35^ | Cases prevented %  Selective serotesting 37 Serotest all 42  Selective vaccination 40  Vaccinate all 45  All options involve vaccination, the first three of susceptibles identified | Reduction in costs: direct (d) indirect (i) %  d i  Selective serotesting 38 37 Serotest all 42 42  Selective vaccination 41 40  Vaccinate all 46 45  Total costs incl programme costs  Selective serotesting 11  Serotest all -  Selective vaccination -11*  Vaccinate all -40*  *ie an increase |  | Incremental cost per QALY gained  All dominated except selective serotesting and vaccinate all  ICER vaccinate all vs serotesting £10,816 | None | 22 | 17 |
| Nettleman  1997^47^ | Not clearly specified | Reduction in cost per person  Serotest then vaccinate susceptibles £24  Vaccinate all £65 | As in cost cell |  |  | 16 | 15 |
| Olson 2001^30^ | Reduction in hospital admissions 95% | Reduction in cost per case 88% | Cost saving see previous cell |  |  | 19 | 15 |
| O’Neill  2003^48^ | Not clearly specified | Reduction in cost over five years 35% | As in cost cell |  |  | 8 | 10 |
| Paternina-Caicedo  2013^52^ | Reduction in cases consultations, hospital admissions, deaths, years of life lost and DALYs %  1 dose 62  2 doses 68 | Change in costs %  Treatment Total  1 dose -60 -67  2 doses 97 247 | Cost per event prevented or LYLST/DALYs gained  One or two doses vs no vaccination; two doses vs one dose (2vs1)  1 2 2vs1  Consult £ 8 18 102  Hosp £k 4.2 9.6 55.1  Death £k 64 147 846  LYG £k 2.2 4.9 28.3  DALY £k 1.1 2.1 15.3 |  |  | 22 | 16 |
| Patrikar 2022^42^ | Changes vs no vaccination  Hospital admissions - 81%  Training days lost - 80% | Unclear | Cost per case averted and cost per man-day saved  Case £3404  Man-day £341 | Cost per QALY £5744 | n/a | 24 | 13 |
| Pawaskar 2021^62^ | Changes vs no vaccination  Cases - 95% to - 96%  Outpatient - 75% to - 85%  Hospital  Admissions - 67% to - 79%  Deaths - 75% to - 79% | See next panel | All strategies save costs and deliver QALY gains vs no vaccination  In order:  Cost: AEFBCD  QALYs: EACFBD  All strategies dominate no vaccination.  First dose 15 months  Second dose  Strategy  AB 7 years  CD 11 years  EF 18 months  AE BF C D  Va Vl Va Vl  Va Vl PQ PT  Va: Varivax  Vl : Varilrix  PQ: ProQuad  PT: Priorix-Tetra | All strategies save costs and deliver QALY gains vs no vaccination  In order:  Cost: EACFBD  QALYs: EACFBD  All strategies dominate no vaccination | Intervention dominant over comparator | 23 | 16 |
| Peña Blasco 2017^75^ | Reduction in cases and treatments:  100% by assumption | Reduction in costs:  100% by assumption | Not reported | Benefit:cost ratio  1.24 | none | 17 | 14 |
| Pinot de Moira  2006^37^ | Reduction in outcomes %  SV HSV  Cases 75 37  during pregnancy 69 22  Hospital  Admissions 100 45  Deaths - 33  CVS in children 69 22  Neonatal varicella 68 21  Reductions similar by country of birth, UK or Bangladesh:  HSV – history taking then serotesting -ves then vaccinating susceptibles | Change in costs %  UK born  SV HSV  Treatment 86 65  Total 166 23  Bangladesh born  SV HSV  Treatment 81 50  Total 49 16 | See cost cell |  | A varicella infection during pregnancy would need to result in an average QALY loss of 0.51 | 19 | 12 |
| Preblud  1985^96^ | Reduction in cases: 78% | Reduction in costs %  Direct medical and  home care 77 Total cost including  vaccination costs 66 | Benefit:cost ratio: 0.3:1 | Benefit:cost ratio: 6.9:1 |  | 12 | 13 |
| Rafferty 2021^55^ | Changes vs no vaccination  Strategy A B  Cases - 95% - 95%  Hospital  Admissions - 95% - 94%  A: 12 and 18 months  B: 12 months and 4-6 years | Changes  Strategy A B  Healthcare 35% - 16%  Societal 33% - 17% | Cost per QALY vs no vaccination or vs other strategy  A £76.060  B £98,841  A vs B £16,972 | Cost per QALY vs no vaccination or vs other strategy  A dominates  B dominates  A vs B £2995 | Deemed not cost-effective from healthcare perspective. | 23 | 17 |
| Sauboin 2012^85^ | Annual number of cases saved over 5 years: 677,738 | “Significant” savings, value not reported. | £14,866 per QALY | Not reported | none | 19 | 14 |
| Scuffham 1999^76^ | 80% coverage vs 10% (base)  Reduction in outcomes %  Cases 72  GP visits 72  Hospital admissions 68  Major complications 68  Outpatient visits 69  LT disability 73  Death 70 | 80% coverage vs 10% (base)  Change in costs %  Treatment -71  Vaccination 716  Healthcare 200  Work loss -67  Total -26 | Benefit:cost ratio 0.67 | Benefit:cost ratio 2.79 |  | 20 | 14 |
| Scuffham  2000^77^ | Reductions in outcomes %  V HV V+cu*  Cases 61 3 3  GP visits 63 9 14  Hospital  admissions 50 11 12  Complics  encephalitis 57 9 11  pneumonia 34 21 23  LT disability 57 10 11  Death 50 15 16  *compared with V  V - vaccinate all  HV – take history and vaccinate susceptibles  V+cu – vaccinate all plus catch up | Change in costs %  VH V V+cu*  Treatment -53 -6 -8  Direct 156 51 9  *compared with V | Cost per event prevented  V HV V+cu*  Case £ 59 483 381  Hospital  Admission  £k 19.6 31.9 23.9  Death £m 9.6 11.1 8.3  *compared with V |  |  | 19 | 16 |
| Smith  1998^36^ | Reduction in outcomes %  HSV SV  Adults  Cases 43 57  Hosp 65 86  Deaths 65 86  Children  CVS 44 58  varicella 43 57  LT disability 46 62  Deaths 43 58  V: vaccinate  H: history of varicella  S: serotest  Sequence: eg  HSV: H then apply S to H- then vaccinate the S-. | Change in costs %  HSV SV  Medical 119 442  Indirect -53 -70  Total -16 41 | ICER Discounted cost per case etc prevented or life year gained  HSV SV  Adult case £ 1272 3566  Fetal case £k 472 1359  Hospital  admission £k 24 66  Death £k 364 10216  LYG* £k 6.6 18.4  *Not discounted  ICER of SV vs V not reported | ICER Discounted cost per case etc prevented or life year gained  HSV^§^ SV  Adult case £ 1528  Fetal case £k 582  Hospital  admission £k 28  Death £k 438  LYG* £k 7.9  §HSV dominates  *Not discounted  ICER of SV vs V not reported |  | 21 | 15 |
| Smith 2000^28^ | Incremental gain in quality adjusted hours  SV vs no V V vs SV  20-29 16.6 0.17  30+ 2.6 0.023  SV – serotest then vaccinate susceptibles  V – vaccinate all | Change in cost %  Payer  SV vs no V V vs SV  20-29 38 91  30+ 787 93  Societal  SV vs no V vs SV  20-29 -63 83  30+ 123 92 | Incremental cost per QALY  SV vs no V V vs SV  20-29 £7200 £2.34m  30+ £167,400 £17m | Incremental cost per QALY  SV vs no V V vs SV  20-29 * £2.3m  30+ £104,800 £17m  *dominates |  | 21 | 15 |
| Thiry  2004^26^ | Reduction in events vs no vaccination %  V HV SV HSV P  Cases 45 34 38 33 17  Hospital  admissions 60 45 51 44 23  Encephalitis 67 50 57 49 25  Deaths 67 49 57 48 24  Life-years  lost 67 50 57 49 25  V – vaccinate  H - take history first  S – serotest first  HS – take history and serotest -ves  P – private | Change in costs vs no vaccination  V HV SV HSV Direct  Medical 254 39 74 22 82  Indirect -34 -39 -25 -34 -19  Total 17 -25 -7 -24 -1 | Benefit:cost ratio vs no vaccination  V HV SV HSV P  0.20 0.54 0.42 0.68 0.22 | Benefit:cost ratio vs no vaccination  V HV SV HSV P  0.78 2.17 1.16 2.171.05 |  | 22 | 16 |
| Thompson 2012^86^ | Not reported | Not reported | The results vary by denominator  (a) the entire population cumulatively over time.  (b) the entire population in the year when steady state attained.  (c) the lifetime of the first birth cohort  Cost per QALY  (a) (b) (c)  £1674 £1033 £3187 | Not reported | none | 21 | 16 |
| Tseng 2005^9^7 | Reduction in outcomes: 80% coverage vs base (10% private) %  Cases 61  Hospital admissions 55 | Change in costs:  80% coverage vs base (10% private) %  Direct medical 86  Direct medical  plus indirect -31 | Cost per outcome prevented: 80% coverage vs base (10% private)  Cases £ 74  Hospital  admissions £ 7812  Benefit:cost ratio 0.36 | Cost per outcome prevented: 80% coverage vs base (10% private)  Cases £ -125  Hospital  admissions £ -3,180  Benefit:cost ratio 1.44 |  | 18 | 14 |
| Valentim 2008^103^ | Change in events over 30 years %  Cases 85  Deaths 81  Life years lost 82  Neurological  sequelae 86 | Change in cost over 30 years %    Healthcare Societal  Varicella -85 -80  Vaccination 2052 2052  Total 459 245 | Cost per event avoided £  Cases 26  Deaths 639,013  Life-year saved 11,636 | Cost per event avoided £  Case 23  Death 576,096  Life-year saved 10,490 | Cost per life year saved less than twice Brazil GDP per head. | 21 | 14 |
| Valentim 2009^102^ | None, only costs change | Societal costs increased by 11%; cost savings increased by 16%. | none | Cost per life year saved fell by 4% to €4098 (£3893) per life year saved. | Cost per life year saved less than thrice Brazil GDP per head. | 15 | 11 |
| Van Hoek 2012^56^ | Reduction in cases vs no vaccination %  Strategy 1 2 3  V 89 0 89  HZ 0 2 3  Discounted QALYs lost  Strategy none 1 2 3  V 60.39 7.76 60.22 7.66  HZ 588 630 562 601  Total 648 638 622 609  1. Childhood varicella (V) 2 doses  2. Elderly herpes zoster (HZ) 1 dose  3. 1&2 | Change in discounted costs infinite time horizon %  Strategy 1 2 3  V -89 0 -89  HZ 0 -2 -3  Net vaccination costs* £m  1 2 3  444 593 1024  *costs of vaccination minus treatment costs averted | Simulations meeting £20k and 30K thresholds  %  vs no vaccination  1 2 3  20k 41 49 50  30k 50 96 70  vs option 1  2 3  20k 61 70  30k 64 99 | none | As in healthcare box | 22 | 17 |
| Van Lier 2015^87^ | Not clearly stated. | Not clearly stated |  | With immune boosting, vaccination at 95% coverage is not cost-effective (threshold €20k per QALY) | As in societal box | 18 | 18 |
| Wang 2021^57^ | Changes vs no vaccination  Strategy A B C D  Cases - 70% - 57% - 92% -76%  Strategy A B C D  Doses 1 1 2 2  Universal U  or selective S U S U S | Changes in cost  Strategy  A B C D  155% 425% 341% 681% | n/a | Cost per QALY vs no vaccination (none) or strategy A  none A  A £16,051  B £31,968 £74,245  C £26,566 £58,662  D £38,555 £65,756 | Strategy A satisfies the three times GDP per head threshold. | 22 | 16 |
| Wolff 2021^88^ | Changes vs no vaccination  Not reported | Change in costs vs no vaccination (varicella only)  Healthcare and societal  - 77% | Not reported | Cost per QALY vs no vaccination  Dominates | Intervention dominates | 23 | 17 |
| Wolfson 2019^53^ | Reduction in events over 100 years %  Strategy (a) (b) (c)  Deaths 94 97 96  Cases 97 98 98  1 dose at 12 months (a)  Repeat dose at 18 months (b) or 6 years (c). | Change in discounted cumulative costs per capita after 100 years with external boosting and herpes zoster %  (a) (b) (c)  Direct -1 -2 -2  Indirect -40 -49 -42  Total -26 -26 -22 | Not reported | Cost per QALY at 100 years  (b) dominates no vaccine and (a). Its cost effectiveness vs (c) is 38,000 TRY (£24,320) /QALY. | (b) satisfies the threshold of 56,600 TRY, Turkey’s GDP per head. | 23 | 17 |
| Zhou 2005^78^ | Reduction in lifetime events in birth cohort %  Cases 83  Deaths 70 | Change in lifetime costs in birth cohort %  Direct -84  Indirect -84  Total -84 | Not reported separately for varicella | Not reported separately for varicella | none | 20 | 16 |
| Zhou 2014^79^ | Reduction in cases: 97 % | Costs saved over lifetime of birth cohort of 4,261,494 £m  Direct 321  Indirect 1374 | Not reported separately for varicella | Not reported separately for varicella | none | 20 | 16 |

**References**

1. Arvin AM. Varicella-zoster virus. Clin Microbiol Rev 1996;9(3):361-381.

2. Public Health England. The Green Book (Chapter 34). London: Public Health England, 2013.

3. Brisson M, Edmunds WJ. Epidemiology of varicella-zoster virus in England and Wales. J Med Virol 2003;70 Suppl 1:S9-14.

4. Walker JL, Andrews NJ, Mathur R, et al. Trends in the burden of varicella in UK general practice. Epidemiol Infect 2017;145(13):2678-2682.

5. Bernal JL, Hobbelen P, Amirthalingam G. Burden of varicella complications in secondary care, England, 2004 to 2017. Euro Surveill 2019;24(42).

6. Kauffmann F, Bechini A, Bonanni P, et al. Varicella vaccination in Italy and Germany - different routes to success: a systematic review. Expert Rev Vaccines 2020;19(9):843-869.

7. Varicella and herpes zoster vaccines: WHO position paper, June 2014. Wkly Epidemiol Rec 2014;89(25):265-287.

8. Marin M, Marti M, Kambhampati A, et al. Global varicella vaccine effectiveness: a meta-analysis. Pediatrics 2016;137(3):e20153741.

9. Shapiro ED, Vazquez M, Esposito D, et al. Effectiveness of 2 doses of varicella vaccine in children. J Infect Dis 2011;203(3):312-315.

10. Annunziato P, Gershon AA. Primary vaccination against varicella. In: Arvin A, Gershon AA eds. Varicella-zoster virus. Cambridge: Cambridge University Press 2000.

11. WHO Introduction of Varicella Vaccination. https://immunizationdata.who.int/pages/vaccine-intro-by-antigen/varicella.html?ISO_3_CODE=&YEAR= Accessed 7 October 2021

12. Varela FH, Pinto LA, Scotta MC. Global impact of varicella vaccination programs. Hum Vaccin Immunother 2019;15(3):645-657.

13. Thiry N, Beutels P, Van Damme P, et al. Economic evaluations of varicella vaccination programmes: a review of the literature. Pharmacoeconomics 2003;21(1):13-38.

14. Rozenbaum MH, van Hoek AJ, Vegter S, et al. Cost-effectiveness of varicella vaccination programs: an update of the literature. Expert Rev Vaccines 2008;7(6):753-782.

15. Unim B, Saulle R, Boccalini S, et al. Economic evaluation of varicella vaccination: results of a systematic review. Hum Vaccin Immunother 2013;9(9):1932-1942.

16. Damm O, Ultsch B, Horn J, et al. Systematic review of models assessing the economic value of routine varicella and herpes zoster vaccination in high-income countries. BMC Public Health 2015;15:533.

17. Liberati A, Altman DG, Tetzlaff J, et al. The PRISMA statement for reporting systematic reviews and meta-analyses of studies that evaluate healthcare interventions: explanation and elaboration. BMJ 2009;339:b2700.

18. https://www.crd.york.ac.uk/prospero/display_record.php?ID=CRD42021249206

19. ‘CCEMG – EPPI-Centre Cost Converter’ (v.1.6 last update: 29 April 2019) https://eppi.ioe.ac.uk/costconversion/default.aspx. Last accessed 30 November 2021.

20. Gomersall JS, Jadotte YT, Xue Y, et al. Conducting systematic reviews of economic evaluations. Int J Evid Based Healthc 2015;13(3):170-178.

21. Siddaway AP, Wood AM, Hedges LV. How to do a systematic review: a best practice guide for conducting and reporting narrative reviews, meta-analyses, and meta-syntheses. Annu Rev Psychol 2019;70:747-770.

22. Drummond M. Evidence-based medicine meets economic evaluation – an agenda for research. In: Donaldson C, Mugford M, Vale L eds. Evidence-based health economics: from effectiveness to efficiency in systematic review. London: BMJ Books 2002.

23. Evers S, Goossens M, de Vet H, et al. Criteria list for assessment of methodological quality of economic evaluations: Consensus on Health Economic Criteria. Int J Technol Assess Health Care. 2005;21(2):240-245.

24. Husereau D, Drummond M, Petrou S, et al. Consolidated Health Economic Evaluation Reporting Standards (CHEERS) statement. Value Health 2013;16(2):e1-5.

25. Lieu TA, Finkler LJ, Sorel ME, et al. Cost-effectiveness of varicella serotesting versus presumptive vaccination of school-age children and adolescents. Pediatrics. 1995;95(5):632-638.

26. Thiry N, Beutels P, Tancredi F, et al. An economic evaluation of varicella vaccination in Italian adolescents. Vaccine. 2004;22(27-28):3546-3562.

27. Hanslik T, Boelle PY, Schwarzinger M, et al. Varicella in French adolescents and adults: individual risk assessment and cost-effectiveness of routine vaccination. Vaccine. 2003;21(25-26):3614-3622.

28. Smith KJ, Roberts MS. Cost effectiveness of vaccination strategies in adults without a history of chickenpox. Am J Med. 2000;108(9):723-729.

29. Kitai IC, King S, Gafni A. An economic evaluation of varicella vaccine for pediatric liver and kidney transplant recipients. Clin Infect Dis. 1993;17(3):441-447.

30. Olson AD, Shope TC, Flynn JT. Pretransplant varicella vaccination is cost-effective in pediatric renal transplantation. Pediatr Transplant. 2001;5(1):44-50.

31. Chui KS, Wu HL, You JH. Cost-effectiveness analysis of varicella vaccine as post-exposure prophylaxis in Hong Kong. Scand J Infect Dis. 2014;46(1):27-33.

32. Boccalini S, Bechini A, Alimenti CM, et al. Assessment of the clinical and economic impact of different immunization protocols of measles, mumps, rubella and varicella in internationally adopted children. Vaccines (Basel). 2020;8(1):01.

33. de Valliere S, Cani N, Grossenbacher M, et al. Comparison of two strategies to prevent varicella outbreaks in housing facilities for asylum seekers. Int J Infect Dis. 2011;15(10):e716-721.

34. Figueira M, Christiansen D, Barnett ED. Cost-effectiveness of serotesting compared with universal immunization for varicella in refugee children from six geographic regions. J Travel Med 2003;10(4):203-207.

35. Merrett P, Schwartzman K, Rivest P, et al. Strategies to prevent varicella among newly arrived adult immigrants and refugees: a cost-effectiveness analysis. Clin Infect Dis. 2007;44(8):1040-1048.

36. Smith WJ, Jackson LA, Watts DH, et al. Prevention of chickenpox in reproductive-age women: cost-effectiveness of routine prenatal screening with postpartum vaccination of susceptibles. Obstet Gynecol. 1998;92(4 Pt 1):535-545.

37. Pinot de Moira A, Edmunds WJ, Breuer J. The cost-effectiveness of antenatal varicella screening with post-partum vaccination of susceptibles. Vaccine. 2006;24(9):1298-1307.

38. Glantz JC, Mushlin AI. Cost-effectiveness of routine antenatal varicella screening. Obstet Gynecol. 1998;91(4):519-528.

39. Burnham BR, Wells TS, Riddle JR. A cost-benefit analysis of a routine varicella vaccination program for United States Air Force Academy cadets. Mil Med. 1998;163(9):631-634.

40. Goh JJ, Ho M, Koh WM, et al. An economic analysis of varicella immunization in the Singapore military. Mil Med Res. 2016;3:3.

41. Howell MR, Lee T, Gaydos CA, et al. The cost-effectiveness of varicella screening and vaccination in U.S. Army recruits. Mil Med. 2000;165(4):309-315.

42. Patrikar S, Bhatti VK, Suryam V, Kotwal A, Basannar DR, Khera A, Kashyap S, Sharma A. Health technology assessment of varicella vaccine in the Armed Forces. Med J Armed Forces India 2022;78(2):213-220.

43. Baracco GJ, Eisert S, Saavedra S, et al. Clinical and economic impact of various strategies for varicella immunity screening and vaccination of health care personnel. Am J Infect Control. 2015;43(10):1053-1060.

44. Chodick G, Ashkenazi S, Livni G, et al. Cost-effectiveness of varicella vaccination of healthcare workers. Vaccine. 2005;23(43):5064-5072.

45. Gayman J. A cost-effectiveness model for analyzing two varicella vaccination strategies. Am J Health Sys Pharm. 1998;55(24 Suppl 4):S4-8.

46. Gray AM, Fenn P, Weinberg J, et al. An economic analysis of varicella vaccination for health care workers. Epidemiol Infect. 1997;119(2):209-220.

47. Nettleman MD, Schmid M. Controlling varicella in the healthcare setting: the cost effectiveness of using varicella vaccine in healthcare workers. Infect Control Hosp Epidemiol. 1997;18(7):504-508.

48. O'Neill J, Buttery J. Varicella and paediatric staff: current practice and vaccine cost-effectiveness. J Hosp Infect. 2003;53(2):117-119.

49. Banz K, Wagenpfeil S, Neiss A, et al. The cost-effectiveness of routine childhood varicella vaccination in Germany. Vaccine. 2003;21(11-12):1256-1267.

50. Damm O, Horn J, Mikolajczyk R, et al. Health economic evaluation of different vaccination strategies against varicella and herpes zoster in Germany. Value Health. 2015;18 (7):A588.

51. Heininger U, Pillsbury M, Samant S, Lienert F, Guggisberg P, Gani R, O’Brien E, Pawaskar M. Health impact and cost-effectiveness assessment for the introduction of universal varicella vaccination in Switzerland. Pediatr Infect Dis J 2021;40:e217–e221.

52. Paternina-Caicedo A, De la Hoz-Restrepo F, Gamboa-Garay O, et al. How cost effective is universal varicella vaccination in developing countries? A case-study from Colombia. Vaccine. 2013;31(2):402-409.

53. Wolfson LJ, Daniels VJ, Pillsbury M, et al. Cost-effectiveness analysis of universal varicella vaccination in Turkey using a dynamic transmission model. PLoS ONE [Electronic Resource]. 2019;14(8):e0220921.

54. Melegaro A, Marziano V, Del Fava E, et al. The impact of demographic changes, exogenous boosting and new vaccination policies on varicella and herpes zoster in Italy: a modelling and cost-effectiveness study. BMC Med. 2018;16(1):117.

55. Rafferty ERS, McDonald W, Osgood ND, Doroshenko A, Farag M. What we know now: an economic evaluation of chickenpox vaccination and dose timing using an agent-based model. Value Health 2021;24(1):50-60.

56. Van Hoek AJ, Melegaro A, Gay N, et al. The cost-effectiveness of varicella and combined varicella and herpes zoster vaccination programmes in the United Kingdom. Vaccine. 2012;30(6):1225-1234.

57. Wang Q, Xiu S, Yang L, Huang J, Cui T, Shi N, Wang X, Shen Y, Chen E, Lu B, Jin H, Lin L. Economic evaluation of varicella vaccination strategies in Jiangsu province, China: a decision-tree Markov model. Hum Vaccin Immunother 2021;17(11):4194-4202.

58. Esmaeeli S, Yaghoubi M, Nojomi M. Cost-effectiveness of varicella vaccination program in Iran. Int J Prev Med. 2017;8:103.

59. Akpo EIH, Cristeau O, Hunjan M, et al. Epidemiological impact and costeffectiveness of varicella vaccination strategies in the United Kingdom (UK). Clin Infect Dis. 2020; 3(11):e3617-e3626.

60. Chacon-Cruz E, Meroc E, Costa-Clemens SA, Clemens R, Verstraeten T. Economic evaluation of universal varicella vaccination in Mexico. Pediatr Infect Dis J 2022;41:439–444.

61. Marijam A, Safonova E, Scherbakov M, Shpeer E, Van Oorschot D, Rudakova A, Tatochenko V, Briko N. Cost effectiveness and budget impact of universal varicella vaccination in Russia. Hum Vaccin Immunother 2022;Mar 8:1-11.

62. Pawaskar M, Burgess C, Pillsbury M, Wisløff T, Flem E. Clinical and economic impact of universal varicella vaccination in Norway: A modeling study. PLoS One 2021 Jul 8;16(7):e0254080.

63. Holl K, Hunjan M, Sauboin C. Pharmacoeconomic evaluation of the introduction of routine varicella vaccination in children in the United Kingdom. Value Health. 2015;18 (7):A583.

64. Brisson M, Edmunds WJ. Varicella vaccination in England and Wales: cost-utility analysis. Arch Dis Child. 2003;88(10):862-869.

65. De La Hoz F, Alvis N, Gamboa O, et al. Cost-effectiveness analysis of the introduction of the varicellavaccine in Colombia. Value Health. 2011;14 (3):A118.

66. Dimitrova M, Zdrakova MM, Faivre P, et al. Estimation of the long-term population costs and benefits for five different varicella childhood immunization strategies in Bulgaria. Value Health. 2018;21 (Supplement 3):S426.

67. Beutels P, Clara R, Tormans G, et al. Costs and benefits of routine varicella vaccination in German children. J Infect Dis. 1996;174 Suppl 3:S335-341.

68. De Boer PT, Postma MJ. Cost-effectiveness of universal vaccination against varicella in the Netherlands. Value Health. 2013;16 (7):A357.

69. Ditkowsky J, Kohlhoff S, Smith-Norowitz TA. The cost-effectiveness of varicella zoster virus vaccination considering late onset asthma. Pediatr Infect Dis J. 2016;35(9):e275-284.

70. Getsios D, Caro JJ, Caro G, et al. Instituting a routine varicella vaccination program in Canada: an economic evaluation. Pediatr Infect Dis J. 2002;21(6):542-547.

71. Gialloreti LE, Divizia M, Pica F, et al. Analysis of the cost-effectiveness of varicella vaccine programmes based on an observational survey in the Latium region of Italy. Herpes. 2005;12(2):33-37.

72. Ginsberg GM, Somekh E. Cost containment analysis of childhood vaccination against varicella in Israel. J Infect. 2004;48(2):119-133.

73. Huse DM, Meissner HC, Lacey MJ, et al. Childhood vaccination against chickenpox: an analysis of benefits and costs. J Pediatr. 1994;124(6):869-874.

74. Lieu TA, Cochi SL, Black SB, et al. Cost-effectiveness of a routine varicella vaccination program for US children. JAMA. 1994;271(5):375-381.

75. Pena Blasco G, Blasco Perez-Aramendia MJ. A cost-benefit analysis of varicella vaccination in Aragon. Arch Argent Pediatr. 2017;115(5):432-438.

76. Scuffham P, Devlin N, Eberhart-Phillips J, et al. The cost-effectiveness of introducing a varicella vaccine to the New Zealand immunisation schedule. Soc Sci Med. 1999;49(6):763-779.

77. Scuffham PA, Lowin AV, Burgess MA. The cost-effectiveness of varicella vaccine programs for Australia. Vaccine. 1999;18(5-6):407-415.

78. Zhou F, Santoli J, Messonnier ML, et al. Economic evaluation of the 7-vaccine routine childhood immunization schedule in the United States, 2001. Arch Pediatr Adolesc Med. 2005;159(12):1136-1144.

79. Zhou F, Shefer A, Wenger J, et al. Economic evaluation of the routine childhood immunization program in the United States, 2009. Pediatrics. 2014;133(4):577-585.

80. Lenne X, Diez Domingo J, Gil A, et al. Economic evaluation of varicella vaccination in Spain: results from a dynamic model. Vaccine. 2006;24(47-48):6980-6989.

81. Azzari C, Baldo V, Giuffrida S, et al. The cost-effectiveness of universal varicella vaccination in italy: a model-based assessment of vaccination strategies. Clinicoecon Outcomes Res. 2020;12:273-283.

82. Bilcke J, van Hoek AJ, Beutels P. Childhood varicella-zoster virus vaccination in Belgium: cost-effective only in the long run or without exogenous boosting? Hum Vaccin Immunother. 2013;9(4):812-822.

83. Littlewood K, Vissers D, Ouwens M, et al. Cost-effectiveness of mass vaccination for varicella in France with MMRV versus MMR. Value Health. 2008;11(6):A433-A433.

84. Littlewood KJ, Scuboin L, Ouwens M. Cost-effectiveness of mass varicella vaccination in France: Economic consequences of an intensive vaccination program. Value Health. 2009;12 (7):A424.

85. Sauboin C, Bonanni P, Ouwens MJ, et al. Pharmacoeconomic evaluation of the introduction of universal varicella vaccination in italy. Value Health. 2012;15 (7):A393.

86. Thompson JR, Talbird SE, Mauskopf JA, et al. Translating outcomes from a dynamic transmission model for varicella vaccination to cost-effectiveness estimates: The impact of different analytic approaches on the results. Value Health. 2012;15 (4):A10.

87. Van Lier A, Lugner A, Opstelten W, et al. Distribution of health effects and cost-effectiveness of varicella vaccination are shaped by the impact on herpes zoster. EBioMedicine. 2015;2(10):1494-1499.

88. Wolff E, Widgren K, Scalia Tomba G, Roth A, Lep T, Andersson S. Cost-effectiveness of varicella and herpes zoster vaccination in Sweden: An economic evaluation using a dynamic transmission model. PLoS One 2021 [Electronic Resource];16(5):e0251644.

89. Coudeville L, Brunot A, Giaquinto C, et al. Varicella vaccination in Italy : an economic evaluation of different scenarios. Pharmacoeconomics. 2004;22(13):839-855.

90. Banz K, Iseli A, Aebi C, et al. Economic evaluation of varicella vaccination in Swiss children and adolescents. Hum Vaccin. 2009;5(12):847-857.

91. Bonanni P, Boccalini S, Bechini A, et al. Economic evaluation of varicella vaccination in Italian children and adolescents according to different intervention strategies: the burden of uncomplicated hospitalised cases. Vaccine. 2008;26(44):5619-5626.

92. Brisson M, Edmunds WJ. The cost-effectiveness of varicella vaccination in Canada. Vaccine. 2002;20(7-8):1113-1125.

93. Diez Domingo J, Ridao M, Latour J, et al. A cost benefit analysis of routine varicella vaccination in Spain. Vaccine. 1999;17(11-12):1306-1311.

94. Hammerschmidt T, Goertz A, Wagenpfeil S, et al. Validation of health economic models: the example of EVITA. Value Health. 2003;6(5):551-559.

95. Hsu HC, Lin RS, Tung TH, et al. Cost-benefit analysis of routine childhood vaccination against chickenpox in Taiwan: decision from different perspectives. Vaccine. 2003;21(25-26):3982-3987.

96. Preblud SR, Orenstein WA, Koplan JP, et al. A benefit-cost analysis of a childhood varicella vaccination programme. Postgrad Med J. 1985;61 Suppl 4:17-22.

97. Tseng HF, Tan HF, Chang CK. Varicella epidemiology and cost-effectiveness analysis of universal varicella vaccination program in Taiwan. Southeast Asian J Trop Med Public Health. 2005;36(6):1450-1458.

98. Blas M, Gutierrez R, Petrozzi V, et al. The cost-effectiveness of varicella vaccination in Peru. Value Health. 2017;20 (9):A942.

99. Coudeville L, Brunot A. Childhood vaccination against varicella in France: An economic evaluation of different strategies. Value Health. 2003;6(6):609-609.

100. Coudeville L, Brunot A, Szucs TD, et al. The economic value of childhood varicella vaccination in France and Germany. Value Health. 2005;8(3):209-222.

101. Coudeville L, Paree F, Lebrun T, et al. The value of varicella vaccination in healthy children: cost-benefit analysis of the situation in France. Vaccine. 1999;17(2):142-151.

102. Valentim J, Sartori A, Amaku M, et al. Cost estimates in the economic evaluations of vaccination programmes: The cases of rotavirus and varicella in Brazil. Value Health. 2009;12 (7):A427.

103. Valentim J, Sartori AM, de Soarez PC, et al. Cost-effectiveness analysis of universal childhood vaccination against varicella in Brazil. Vaccine. 2008;26(49):6281-6291.

104. Sanders GD, Neumann PJ, Basu A, et al. Recommendations for Conduct, Methodological Practices, and Reporting of Cost-effectiveness Analyses: Second Panel on Cost-Effectiveness in Health and Medicine. JAMA. 2016;316(10):1093-1103.

105. Hodgkinson B, Wang T, Byrnes J, et al. Modelling a cost-effective vaccination strategy for the prevention of varicella and herpes zoster infection: A systematic review. Vaccine. 2021;39(9):1370-1382.
